# Harnessing plant-derived extracellular vesicles in advanced biomaterials: from structural scaffolds to responsive systems for next-generation wound therapeutics

**DOI:** 10.1093/burnst/tkag045

**Published:** 2026-07-02

**Authors:** Min Tan, Fengqin Luo, Ya Xu, Yuhe Dai, Junqi Yang, Qianbo Zhang, Xiuying Guo, Lele Liu, Ming Tang, Felicity Han, Xuqiang Nie

**Affiliations:** College of Pharmacy, Key Laboratory of the Basic Pharmacology of the Ministry of Education and Joint International Research Laboratory of Ethnomedicine of Ministry of Education, Zunyi Medical University, No. 6 Xuefu West Road, Xinpu New District, Zunyi, Guizhou 563006, China; Guizhou Key Laboratory of Modern Traditional Chinese Medicine Creation, Zunyi Medical University, No. 6 Xuefu West Road, Xinpu New District, Zunyi, Guizhou 563006, China; College of Pharmacy, Key Laboratory of the Basic Pharmacology of the Ministry of Education and Joint International Research Laboratory of Ethnomedicine of Ministry of Education, Zunyi Medical University, No. 6 Xuefu West Road, Xinpu New District, Zunyi, Guizhou 563006, China; Guizhou Key Laboratory of Modern Traditional Chinese Medicine Creation, Zunyi Medical University, No. 6 Xuefu West Road, Xinpu New District, Zunyi, Guizhou 563006, China; College of Pharmacy, Key Laboratory of the Basic Pharmacology of the Ministry of Education and Joint International Research Laboratory of Ethnomedicine of Ministry of Education, Zunyi Medical University, No. 6 Xuefu West Road, Xinpu New District, Zunyi, Guizhou 563006, China; Guizhou Key Laboratory of Modern Traditional Chinese Medicine Creation, Zunyi Medical University, No. 6 Xuefu West Road, Xinpu New District, Zunyi, Guizhou 563006, China; College of Pharmacy, Key Laboratory of the Basic Pharmacology of the Ministry of Education and Joint International Research Laboratory of Ethnomedicine of Ministry of Education, Zunyi Medical University, No. 6 Xuefu West Road, Xinpu New District, Zunyi, Guizhou 563006, China; Guizhou Key Laboratory of Modern Traditional Chinese Medicine Creation, Zunyi Medical University, No. 6 Xuefu West Road, Xinpu New District, Zunyi, Guizhou 563006, China; College of Pharmacy, Key Laboratory of the Basic Pharmacology of the Ministry of Education and Joint International Research Laboratory of Ethnomedicine of Ministry of Education, Zunyi Medical University, No. 6 Xuefu West Road, Xinpu New District, Zunyi, Guizhou 563006, China; Guizhou Key Laboratory of Modern Traditional Chinese Medicine Creation, Zunyi Medical University, No. 6 Xuefu West Road, Xinpu New District, Zunyi, Guizhou 563006, China; College of Pharmacy, Key Laboratory of the Basic Pharmacology of the Ministry of Education and Joint International Research Laboratory of Ethnomedicine of Ministry of Education, Zunyi Medical University, No. 6 Xuefu West Road, Xinpu New District, Zunyi, Guizhou 563006, China; Guizhou Key Laboratory of Modern Traditional Chinese Medicine Creation, Zunyi Medical University, No. 6 Xuefu West Road, Xinpu New District, Zunyi, Guizhou 563006, China; College of Pharmacy, Key Laboratory of the Basic Pharmacology of the Ministry of Education and Joint International Research Laboratory of Ethnomedicine of Ministry of Education, Zunyi Medical University, No. 6 Xuefu West Road, Xinpu New District, Zunyi, Guizhou 563006, China; Guizhou Key Laboratory of Modern Traditional Chinese Medicine Creation, Zunyi Medical University, No. 6 Xuefu West Road, Xinpu New District, Zunyi, Guizhou 563006, China; College of Pharmacy, Key Laboratory of the Basic Pharmacology of the Ministry of Education and Joint International Research Laboratory of Ethnomedicine of Ministry of Education, Zunyi Medical University, No. 6 Xuefu West Road, Xinpu New District, Zunyi, Guizhou 563006, China; Guizhou Key Laboratory of Modern Traditional Chinese Medicine Creation, Zunyi Medical University, No. 6 Xuefu West Road, Xinpu New District, Zunyi, Guizhou 563006, China; Department of Structural Biology, St. Jude Children’s Research Hospital, 262 Danny Thomas Place, Memphis, TN 38105, United States; Australian Institute for Bioengineering and Nanotechnology, The University of Queensland, Building 75, Corner of College Road and Cooper Road St Lucia, Brisbane, QLD 4072, Australia; College of Pharmacy, Key Laboratory of the Basic Pharmacology of the Ministry of Education and Joint International Research Laboratory of Ethnomedicine of Ministry of Education, Zunyi Medical University, No. 6 Xuefu West Road, Xinpu New District, Zunyi, Guizhou 563006, China; Guizhou Key Laboratory of Modern Traditional Chinese Medicine Creation, Zunyi Medical University, No. 6 Xuefu West Road, Xinpu New District, Zunyi, Guizhou 563006, China; Zunyi Center for Disease Control and Prevention, No. 2 Xinlong Avenue, Xinpu New District, Honghuagang District, Zunyi, Guizhou 563006, China

**Keywords:** Wound healing, Plant-derived extracellular vesicles, Biomaterials, Tissue engineering, Regenerative medicine, Extracellular vesicles, Wound therapy

## Abstract

Chronic non-healing wounds, particularly diabetic foot ulcers, persist as a significant clinical challenge due to the limited availability of therapeutic options and the complexity of their pathological microenvironments. Plant-derived extracellular vesicles (PDEVs), which are nanoscale lipid carriers rich in bioactive molecules, have emerged as a promising therapeutic approach due to their low immunogenicity, antioxidant, anti-inflammatory properties, and their ability to promote angiogenesis. Structurally, the biomaterial protects the integrity of PDEVs and extends their residency through modulated release. Biologically, PDEVs enhance the regenerative potential of the material by profoundly reprogramming the local cellular and immune microenvironments. However, their clinical application is severely impeded by their inherent instability in the dynamic wound microenvironment, which includes susceptibility to enzymatic degradation, disruption due to pH fluctuations, and rapid clearance by wound exudate. These factors collectively lead to premature cargo loss and reduced therapeutic efficacy. This review synthesizes how engineered biomaterials, serving as tailored structural platforms for controlled delivery, can overcome these stability challenges specific to PDEVs. By examining loading strategies and design principles, we demonstrate how composite systems surpass single-component therapies in wound models. Lastly, we address translational challenges and propose a development framework that integrates artificial intelligence–driven design with mechanism-guided material optimization. This work lays the groundwork for the rational development of next-generation vesicle-enabled wound therapeutics.

## Graphical Abstract

**Graphical Abstract:**
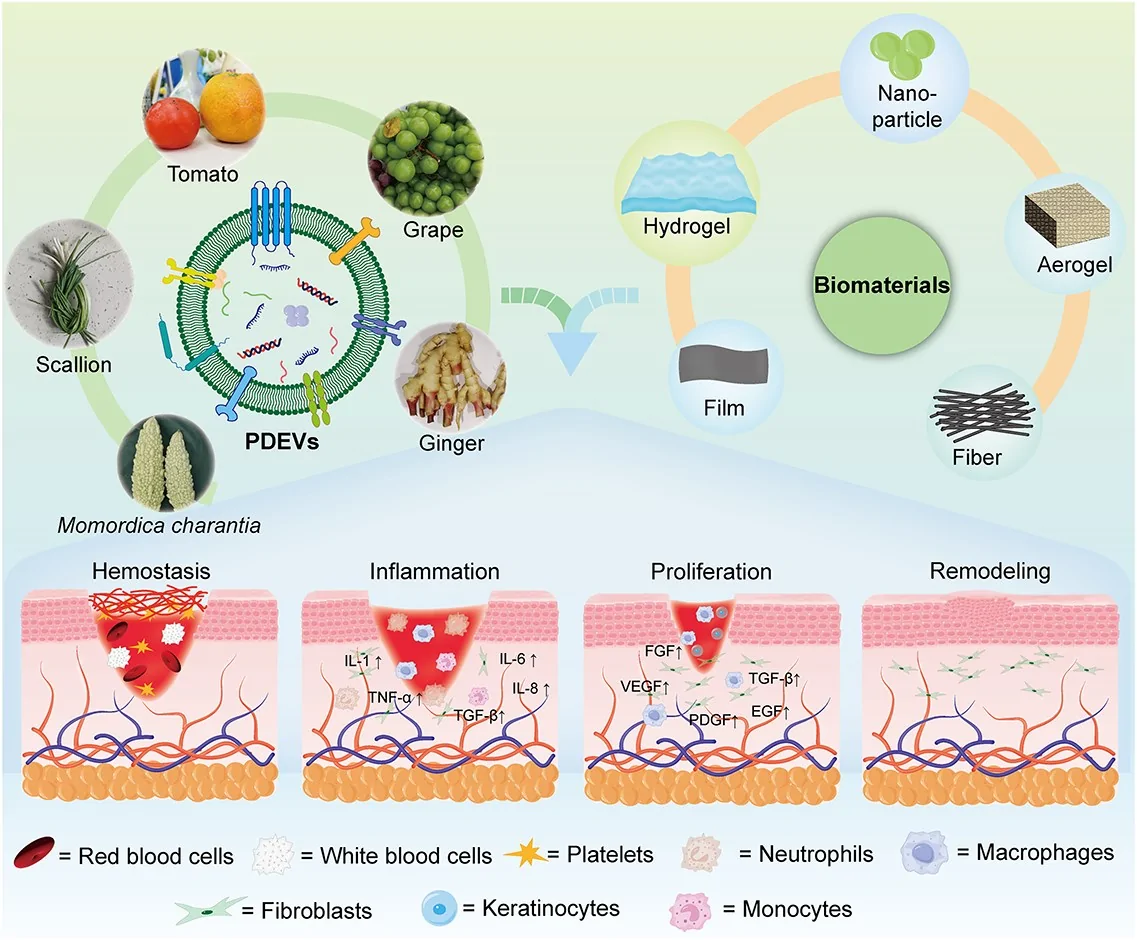

## Highlights

- Plant-derived extracellular vesicles boost wound repair *via* multiple signaling pathways.
- Biomaterial carriers stabilize vesicles and enable controlled therapeutic release.
- Vesicle-biomaterial composites synergistically accelerate wound closure.
- Combinatorial plant vesicle therapies show supra-additive healing efficacy.

## Background

The skin and mucosal surfaces are the body’s primary physical barriers, offering critical protection against environmental insults [1–3]. However, severe or recurrent skin injuries may lead to impaired healing, presenting substantial clinical challenges. Complications such as severe infections, limb amputations, and mortality highlight the urgent need for effective wound management [4, 5].

Clinically, wounds are categorized into acute and chronic types [6]. Acute wounds typically adhere to an orderly healing process and heal within the anticipated timeframe. In contrast, chronic wounds, including diabetic foot ulcers (DFUs), venous leg ulcers, and pressure injuries, are characterized by arrested healing phases, resulting in prolonged recovery times. This delay is typically attributed to underlying diseases or pathological factors that disrupt the healing process. According to the World Health Organization (WHO), chronic wounds affect ~2% of the global population annually, account for 4% of total healthcare expenditures, and constitute ~70% of the nursing workload [7]. They pose a persistent and multifaceted clinical burden.

Wound healing ordinarily progresses through four overlapping yet distinct phases: hemostasis, inflammation, proliferation, and remodeling [8, 9]. Chronic wounds, however, remain in an extended inflammatory phase, creating a hostile microenvironment characterized by sustained oxidative stress, excessive reactive oxygen species (ROS), and compromised cellular function [10, 11]. This enduring inflammation elevates infection risks and impedes tissue proliferation. Thus, effective therapeutic approaches must be comprehensive, simultaneously suppressing inflammation, neutralizing ROS, controlling microbial burden, and promoting cellular proliferation and angiogenesis [12–14].

In recent years, extracellular vesicles (EVs) have emerged as significant players in wound healing, facilitating intercellular communication and regulating physiological and pathological processes by transporting biomolecules such as nucleic acids, proteins, and lipids. Animal-derived EVs, particularly those derived from stem cells, have demonstrated remarkable capabilities in enhancing wound healing [15, 16]. However, they pose a risk of immune rejection from heterologous sources, potential contamination by pathogens, and raise ethical concerns. Conversely, plant-derived extracellular vesicles (PDEVs) are considered favorable candidates for therapeutic applications due to their rich content of bioactive molecules, intrinsic biocompatibility, and potential to act as natural nanocarriers for phytotherapeutic agents [17]. In wound treatment, PDEVs confer dual benefits: regulating key cellular processes such as proliferation, migration, and inflammatory signaling while promoting tissue regeneration [18, 19]. Nevertheless, their direct application *in vivo* is severely hindered by poor stability. The harsh, dynamic conditions of the wound microenvironment, such as fluctuating pH, proteases, and temperature, rapidly degrade PDEVs’ membranes, leading to a premature release of their cargo and reduced therapeutic efficacy [20].

Advanced biomaterials have emerged as essential therapeutic modalities in wound care. Contemporary functional biomaterials overcome the fundamental drawbacks inherent in conventional dressings, such as inadequate moisture regulation, limited antimicrobial properties, and insufficient tissue adhesion [21–23]. Notable examples of advanced systems include hyaluronic acid-based hydrogels [24, 25], chitosan aerogels [26, 27], and films incorporating silver nanoparticles [28, 29]. These engineered platforms foster favorable conditions for modulating cellular behaviors and for the remodeling of microenvironments during wound healing. Additionally, they serve as efficient delivery vehicles for therapeutic agents [30–32].

The integration of PDEVs with advanced biomaterials results in a profound synergistic effect: the biomaterial provides structural protection to the vesicles and enhances the physical closure of wounds, while the PDEVs deliver multi-target biochemical signals. Although previous reviews have discussed PDEVs and biomaterials separately [17, 33, 34], there has been no comprehensive evaluation of their combined platforms. This review aims to address this gap by critically examining the emerging PDEV-biomaterial paradigm. We begin by summarizing the therapeutic mechanisms of PDEVs in wound repair, followed by an assessment of how biomaterial strategies enhance the stability and bioavailability of PDEVs. We conclude by synthesizing the current evidence concerning these composite systems and identifying key priorities for future clinical translation.

## Review

### Overview of PDEVs

**Figure 1:**
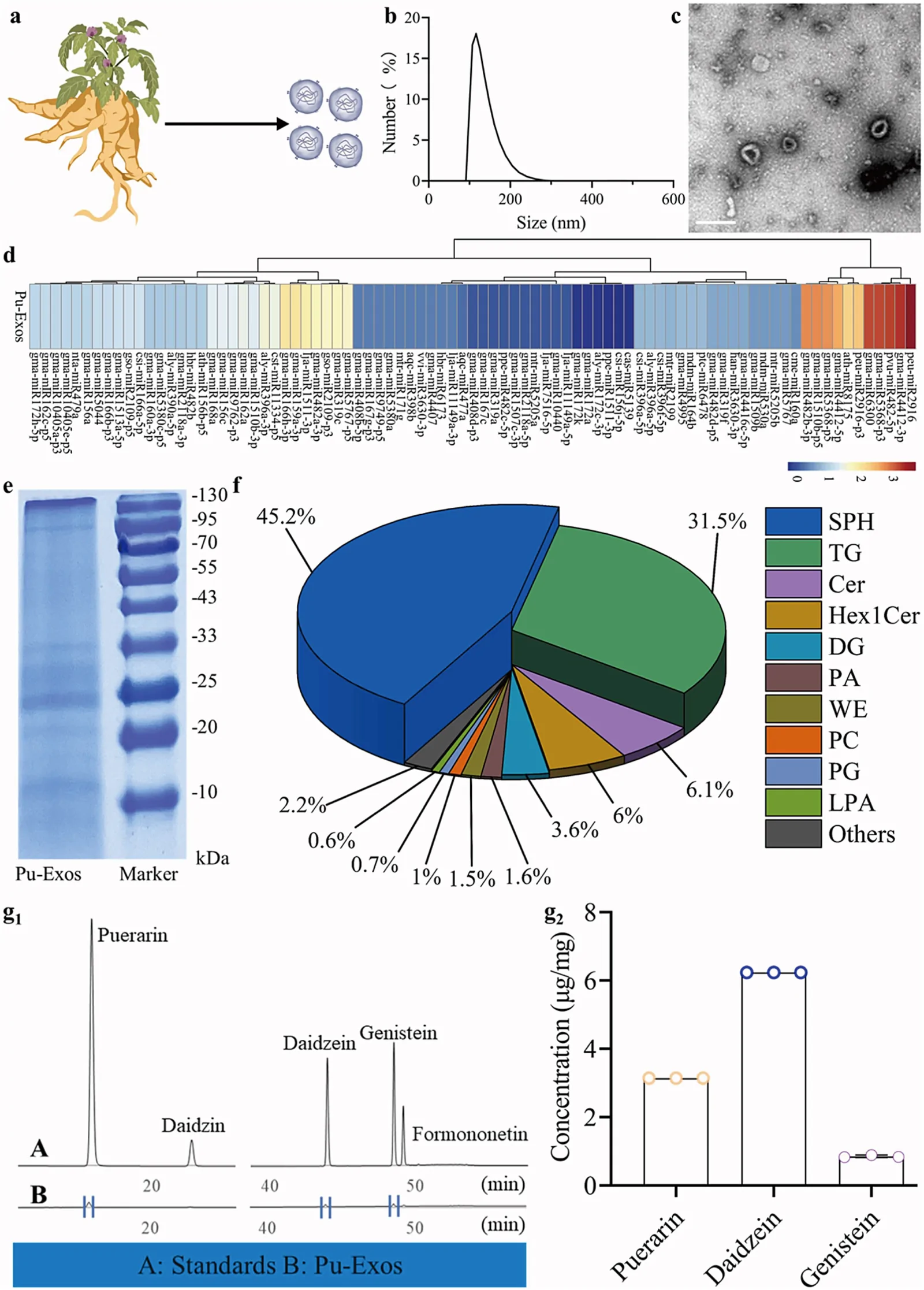
Preparation and characterization of PDEVs derived from *Pueraria lobata* (Pu-Exos). (**a**) Schematic diagram of the preparation process of Pu-Exos. (**b**) Size distribution of Pu-Exos. (**c**) TEM image of Pu-Exos. Scale bar: 200 nm. (**d**) Heatmap of the Pu-Exos-miRNAs. (**e**) The molecular weights distribution of proteins in Pu-Exos. (**f**) Category and quantities of the lipids in Pu-Exos. (**g1**) HPLC of several small molecules in Puerariae Lobatae and Pu-Exos. (**g2**) Quantitative analysis of small molecules in Pu-Exos [35]. Copyright 2024, Elsevier. *PDEVs* plant-derived extracellular vesicles, *Pu-Exos* Pueraria lobata, *TEM* transmission electron microscopy, *HPLC* high-performance liquid chromatography, *WE* wax esters, *PG* phosphatidylglycerol, *LPA* lysophosphatidic acid

PDEVs are nanoscale vesicles with diameters typically ranging from 30 to 300 nm. These vesicles exhibit a spherical or cup-shaped morphology and are secreted through endosomal or alternative pathways. Composed primarily of proteins, lipids, and nucleic acids, PDEVs are illustrated in Figure 1, which presents representative transmission electron microscopy (TEM) images and size distribution data for *Pueraria lobata*-derived PDEVs, depicting their typical nanoscale morphology and structural characteristics [35]. The unique lipid bilayer architecture of PDEVs suggests they may play crucial roles in intercellular communication and physiological regulation [36, 37]. The concept of PDEVs was first entertained in 1967 when Halperin *et al*. identified multivesicular bodies (MVBs) in carrot cell cultures, which exhibited vesicle-like architecture and activity [38]. In a significant development in 2009, de la Canal’s team identified phospholipid components, phosphatidic acid (PA) and phosphatidylinositol, in the extracellular fluid of sunflower seeds. This discovery was later extended to tomato extracellular fluids, suggesting that, akin to animal cells, plant cells might also produce EV-like structures. Working from this premise, the group successfully isolated vesicles from sunflowers using centrifugation methods initially developed for animal vesicles, marking the first successful isolation of PDEVs [39].

PDEVs are structurally analogous to animal EVs and offer several advantages for therapeutic development. These include: (i) derivation from sources that are inherently free of zoonotic or human pathogens, which results in a favorable safety profile and low immunogenicity [40, 41]; (ii) sourcing from abundant and renewable botanical biomass, which supports scalable production [42]; (iii) the capacity to carry evolutionarily conserved cargo molecules, such as miRNAs, facilitating cross-kingdom regulation [43]; and (iv) intrinsic enrichment with anti-inflammatory, antioxidant, and immunomodulatory phytochemicals that provide direct therapeutic benefits [44, 45].

Currently, PDEVs have been consistently isolated from a diverse array of botanical sources. These include vegetables such as *Momordica charantia* [46–48], garlic [49], and onion [50]; fruits such as apples [51], blueberries [52], and grapefruit [53]; and medicinal plants like *Pueraria lobata* [35], *Artemisia annua* [54], and *Davallia Rhizome* [55, 56]. These vesicles retain the molecular signatures of their tissues of origin, enabling them to mediate a broad spectrum of physiological processes effectively [57].

### Activity of PDEVs in wound healing

**Figure 2:**
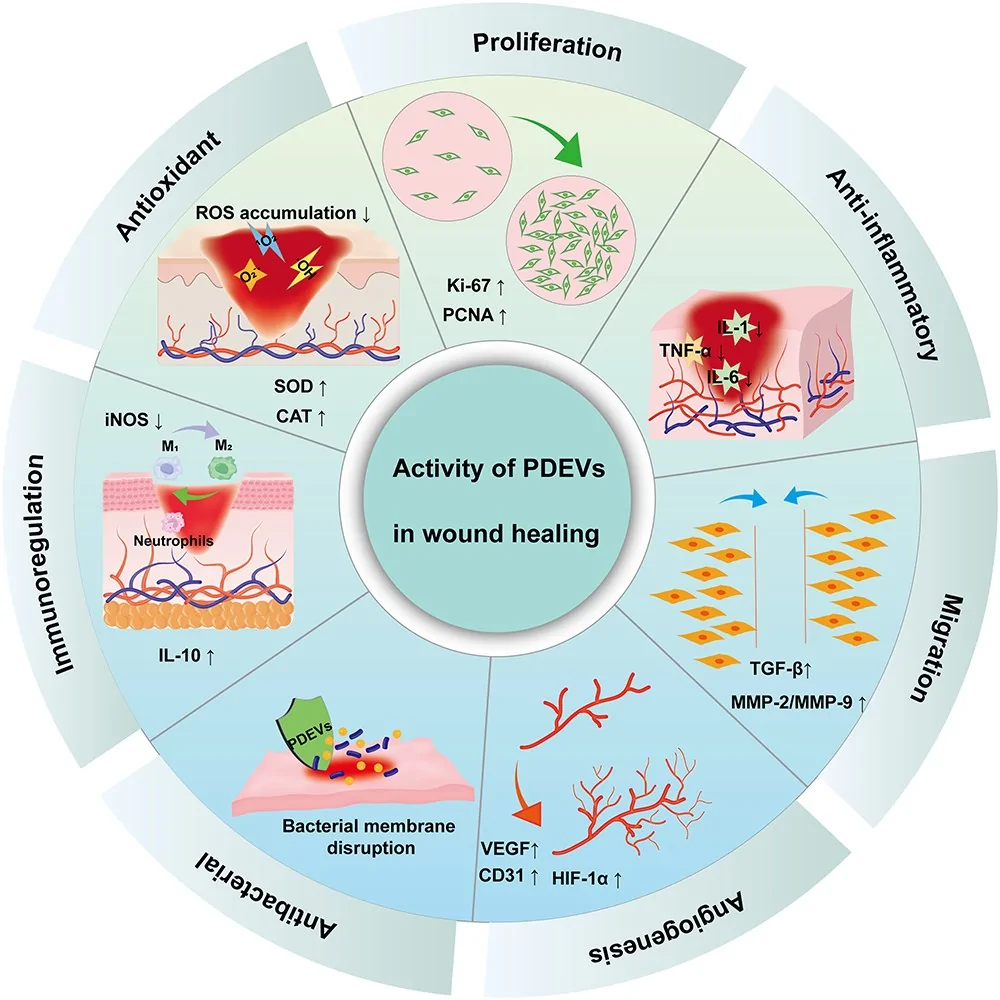
The biological activities of PDEVs. The common biological activities of PDEVs in wound healing include proliferation, anti-inflammation, migration, angiogenesis, antibacterial activity, immunoregulation, and antioxidation. These biological activities are mediated by regulating relevant cellular processes and modulating the expression of specific biomarkers. Arrows indicating their upregulation or downregulation. *ROS* reactive oxygen species, *SOD* superoxide dismutase, *iNOS* inducible nitric oxide synthase, PDEVs plant-derived extracellular vesicles, *VEGF* vascular endothelial growth factor, *HIF-1α* hypoxia-inducible factor 1 alpha, *TNF* tumor necrosis factor, *IL* interleukin, *TGF-β* transforming growth factor beta, *PCNA* proliferating cell nuclear antigen, *MMP* matrix metallopeptidase, *CAT* catalase, *CD31* platelet endothelial cell adhesion molecule-1

Wound healing is a complex physiological process that involves coordinated interactions among various cell types, cytokines, and signaling pathways. Enriched with a multiplicity of bioactive molecules, PDEVs exhibit antioxidant, anti-inflammatory, and proliferative activities that can accelerate repair processes (Figure 2). To provide a systematic framework for understanding the effects mediated by PDEVs, we have organized the key mechanistic signaling axes, their molecular components, validated PDEV sources, and evidence maturity levels in Table 1 [58–75]. This mechanistic stratification offers several critical insights: (i) the Nrf2-ARE antioxidant and NF-κB anti-inflammatory pathways are the most robustly validated, with multiple sources of PDEVs demonstrating consistent therapeutic effects; (ii) the MAPK/YAP1 migration and PI3K/Akt proliferation pathways present emerging evidence, which requires further validation across different sources; and (iii) metabolic reprogramming through the upregulation of glycolytic enzymes represents a novel preliminary finding that warrants priority investigation. This evidence-based framework enables researchers to identify established therapeutic targets and explore promising avenues for future mechanistic research.

**Table 1:** Summary of key mechanistic signaling axes mediating PDEV bioactivities

Mechanistic axis	Core signaling pathway	Key signaling components and molecular targets	PDEV sources with validated effects	Function	Validation level	Key references
**Nrf2 antioxidant axis**	Nrf2/Keap1/HO-1 pathway	NQO1↑, HO-1↑, Nrf2↑, Keap1↓, MDA↓, GSH↑, SOD↑	*Aloe vera* peel, ginseng, *Ulva lactuca*, lemon, shallot and garlic, *Solanum lycopersicum*	ROS scavenging; oxidative stress attenuation; cytoprotection against oxidative damage; enhanced cellular antioxidant capacity	Multi-study validated	Kim *et al*. [58]; Li *et al*. [59]; Li *et al*. [60]; Gasparro *et al*. [61]; Kalarikkal *et al*. [62]; Shen *et al*. [63]
**NF-κB anti-inflammatory axis**	ERK/NF-κB pathway	TNF-α↓, IL-1β↓, IL-6↓, COX2↓, iNOS↓, NF-κB↓, TLR4↓	Ginseng, lemon, *Catharanthus roseus*, *Portulaca oleracea* L*.*	Suppression of pro-inflammatory cytokine release; M1 → M2 macrophage polarization; reduced inflammatory cell infiltration; accelerated inflammation resolution	Multi-study validated	Li *et al*. [59]; Raimondo *et al*. [64]; Ou *et al*. [65]; Long *et al*. [66]
**MAPK cascade axis**	MAPK/ERK/MEK pathway	MEK1/2↑, ERK1/2↑, p38↓	Ginseng, *Lycium ruthenicum* Murray, *Morinda officinalis*, *Taraxacum mongolicum* Hand.-Mazz*.*	Promotion of cell proliferation and migration; alleviation of inflammatory damage; induction of angiogenesis	Multi-study validated	Zhang *et al*. [67]; Zhao *et al*. [19]; Wang *et al.* [68]; Sun *et al*. [69]
**PI3K/Akt proliferation axis**	PI3K/Akt/mTOR pathway	p-PI3K/PI3K↑, p-Akt/Akt↑, p-mTOR/mTOR↑, p-4EBP1/4EBP1↑， p-p70S6K/p70S6K ↑	Gouqi	Stimulated cell viability, increased ALP activity, and promoted cellular osteogenic protein expression	Emerging evidence	Xu *et al*. [70];
**VEGF/VEGFR2 angiogenesis axis**	VEGF/VEGFR2 pathway	VEGF↑, VEGFR2↑, CD31↑	*Dendrobium officinale*, *Epimedium brevicornu* Maxim	Endothelial cell tube formation; neovascularization; capillary network density increase; enhanced blood supply to wound bed	Multi-study validated	Tu *et al*. [71]; Cai *et al*. [72]
**Akt/eNOS vascular axis**	Akt/eNOS/NO pathway	p-Akt/Akt ↑, p-eNOS/eNOS ↑, NO ↑, VEGFR2↑	*Dendrobium officinale*	Vasodilation; endothelial function improvement; angiogenesis promotion; enhanced microcirculation	Emerging evidence	Tu *et al*. [71];
**PI3K/Akt/MAPK pro-angiogenic axis**	PI3K/Akt/MAPK/HMOX1 pathway	PI3K↑, Akt↑, HMOX1↑ ERK1/2↑	*Salvia miltiorrhiza* roots	Vascular endothelial cell angiogenic capacity enhancement; tube formation; endothelial migration	Emerging evidence	Huo *et al*. [73]
**TGF-β/Smad remodeling axis**	TGF-β/Smad2/3 pathway	TGF-β↑, p-Smad2/3↑, COL-1↑, ALP↑	Lotus root	Collagen synthesis and organization; ECM remodeling	Emerging evidence	Xi *et al*. [74];
**PPAR metabolic axis**	PPAR signaling pathway	PFKM↑， PGK1↑， ENO1↑， ACACA↑， PDHA1↑	Ginseng	Anti-inflammatory effects *via* PPAR activation	Emerging evidence	Tan *et al*. [75]
**Glycolytic reprogramming axis**	Glycolysis/HIF-1α pathway	PFKM↑, PGK1↑, ENO1↑, HIF-1α↑, glucose uptake↑, lactate↑	Ginseng	Cellular energy metabolism enhancement; support for proliferating cells; Warburg effect modulation in wound healing	Emerging evidence	Tan *et al*. [75]

Bold text indicates the specific mechanistic signaling axes, and italicized text represents the scientific (Latin) names of the plant species. The arrows (↑ and ↓) indicate the upregulation and downregulation of the respective signaling components and targets. **Validation level criteria**: **multi-study validated** requires ≥3 independent publications from ≥2 research groups, with evidence spanning both *in vitro* cellular assays and *in vivo* wound models. **Emerging evidence** denotes <3 publications or evidence limited to a single experimental modality. **Preliminary finding** indicates a single study with initial *in vitro* observations only. *Nrf2* nuclear factor erythroid 2-related factor 2, *Keap1* Kelch-like ECH-associated protein 1, *HO-1* heme oxygenase 1, *NQO1* NAD(P)H quinone dehydrogenase 1, *MDA* malondialdehyde, *GSH* glutathione, *SOD* superoxide dismutase, *NF-κB* nuclear factor kappa B, *ERK* extracellular regulated kinase, *TNF-α* tumor necrosis factor alpha, *IL* interleukin, *COX2* cyclooxygenase 2, *iNOS* inducible nitric oxide synthase, *TLR4* toll-like receptor 4, *MAPK* mitogen-activated protein kinase, *MEK* MAPK/ERK kinase, *PI3K* phosphatidylinositol 3-kinase, *Akt* protein kinase B, *mTOR* mammalian target of rapamycin, *4EBP1* eukaryotic translation initiation factor 4E binding protein 1, *p70S6K* p70 ribosomal S6 kinase, *VEGF* vascular endothelial growth factor, *CD31* platelet endothelial cell adhesion molecule 1, *eNOS* endothelial nitric oxide synthase, *HMOX1* heme oxygenase 1, *TGF-β* transforming growth factor beta, *Smad2/3* Smad family member 2/3, *COL-1* collagen type I, *ALP* alkaline phosphatase, *PPAR* peroxisome proliferator-activated receptor, *PFKM* phosphofructokinase muscle type, *PGK1* phosphoglycerate kinase 1, *ENO1* enolase 1, *ACACA* acetyl-CoA carboxylase alpha, *PDHA1* pyruvate dehydrogenase E1 alpha subunit, *HIF-1α* hypoxia-inducible factor 1 alpha, *ECM* extracellular matrix

#### Cargo-driven mechanisms of PDEVs in wound healing

The diverse therapeutic effects of PDEVs are fundamentally determined by their distinct, source-specific molecular cargo, primarily comprising micro ribonucleic acids (miRNAs), proteins, lipids, and bioactive metabolites. Understanding the individual and synergistic roles of these components is crucial. PDEVs are highly enriched in small non-coding RNAs that facilitate cross-kingdom regulation. Following endocytosis by mammalian cells, plant-derived miRNAs can execute post-transcriptional gene silencing, downregulating pro-inflammatory signaling cascades or upregulating angiogenic pathways [76]. The protein cargo of PDEVs often includes antioxidant enzymes, such as superoxide dismutase, catalase, and ascorbate peroxidase, and structural proteins that provide immediate direct radical scavenging capabilities at the wound site, rapidly reducing the oxidative burden even before the activation of endogenous mammalian pathways [77]. The lipid bilayer of PDEVs not only facilitates efficient membrane fusion and cellular uptake but also contains bioactive lipids, such as PAs, that are involved in intracellular signaling. Additionally, PDEVs encapsulate concentrated secondary metabolites, including flavonoids, phenolic acids, and alkaloids, which act as potent anti-inflammatory agents and metabolic regulators [78]. Collectively, this multi-omic cargo orchestrates the complex mechanisms of wound repair discussed below.

#### Antioxidant and anti-inflammatory effects

Upon the formation of a wound, the body naturally initiates an inflammatory response as a defensive mechanism. However, excessive or sustained inflammation can lead to further tissue damage. During this phase, immune cells release pro-inflammatory mediators such as interleukin-1 (IL-1) and tumor necrosis factor-α (TNF-α). These cytokines induce vasodilation and increase vascular permeability, which clinically presents as erythema, edema, and pain [79, 80]. Simultaneously, infiltrating leukocytes produce ROS and reactive nitrogen species (RNS). Dysregulation of these species can trigger oxidative stress, leading to the oxidative damage of lipids, proteins, and nucleic acids [81]. Therefore, effective wound therapeutics must often rebalance inflammatory signaling while mitigating oxidative injury.

In diabetic wound therapy, the persistently elevated glucose levels in the local microenvironment present a significant and complex barrier to healing. Hyperglycemia promotes the accumulation of advanced glycation end products (AGEs), which activate the receptor for AGEs (RAGE) on endothelial cells, macrophages, and fibroblasts, thereby sustaining NF-κB-mediated pro-inflammatory signaling. Concurrently, elevated glucose levels stimulate excessive ROS production through mechanisms such as mitochondrial electron transport chain overload, activation of the polyol pathway, stimulation of the protein kinase C pathway, and flux through the hexosamine biosynthetic pathway. This sequence of events forms a detrimental cycle involving hyperglycemia, AGE accumulation, RAGE activation, NF-κB-driven inflammation, impaired Nrf2-ARE antioxidant defense, persistent oxidative stress, and further cellular dysfunction. Additionally, high glucose levels impair macrophage phagocytic function, delay the transition from M1 to M2 macrophage phenotypes, suppress fibroblast proliferation and migration, and inhibit angiogenesis mediated by endothelial progenitor cells. Given these challenges, the robust antioxidant capacities of PDEVs are invaluable for interrupting this hyperglycemic inflammatory cycle.

PDEVs encapsulate antioxidant enzymes, such as superoxide dismutase and catalase, as well as anti-inflammatory phytochemicals including flavonoids and phenolic acids. Studies have demonstrated that PDEVs can attenuate oxidative stress through direct radical scavenging and by reducing the expression of pro-inflammatory cytokines [82], thereby restoring redox homeostasis and promoting the resolution of inflammation.

The Nrf2 and NF-κB pathways are the two most extensively validated signaling axes that govern the antioxidant and anti-inflammatory effects mediated by PDEVs [61, 64, 65]. *Aloe vera* peel-derived PDEVs predominantly exert their antioxidant effects through the activation of the Nrf2 pathway [58]. Similarly, ginseng-derived PDEVs have been shown to alleviate oxidative stress by activating the Nrf2/HO-1 signaling pathway and to mitigate inflammatory responses by inhibiting the TLR4/NF-κB signaling pathway [59]. Importantly, the modulation of these antioxidant and anti-inflammatory pathways by PDEVs is not only parallel but also deeply interconnected through complex molecular crosstalk. PDEVs activate the Nrf2 pathway, which subsequently upregulates antioxidant enzymes such as HO-1. These enzymes scavenge excess ROS, and the consequent reduction in oxidative stress prevents the phosphorylation of IκB kinase (IKK). In the absence of IKK activity, NF-κB remains sequestered in the cytoplasm, unable to translocate to the nucleus to promote pro-inflammatory gene expression [83]. In addition, metabolic byproducts of HO-1, such as carbon monoxide and biliverdin, directly suppress NF-κB activity, thereby exerting anti-inflammatory effects [84]. Conversely, excessive activation of NF-κB can suppress the Nrf2-ARE pathway by competing for co-activators such as p300/CREB [85]. This reciprocal regulation between Nrf2 and NF-κB suggests that PDEVs capable of robustly activating the Nrf2 pathway may also effectively achieve anti-inflammatory outcomes, thus offering a dual-target therapeutic strategy with a single molecular intervention.

Further studies have identified additional PDEVs that regulate the Nrf2 and NF-κB signaling pathways, such as those derived from *Ulva lactuca* [60], *Solanum lycopersicum* [63], shallot, and garlic [62]. These findings not only further validates the Nrf2 and NF-κB pathways as core regulatory targets of PDEVs but also suggest that such regulatory mechanisms may be widely conserved across different PDEVs [59, 66]. Additional PDEVs demonstrating notable antioxidant and anti-inflammatory capabilities are derived from *Opuntia ficus-indica* fruit [86], *Lithospermum erythrorhizon* callus [87], pomegranate [88], and prickly pear fruit juice [89], though their precise mechanistic pathways require further investigation.

Beyond the applications of single-source products, the combination of PDEVs from multiple sources may synergistically enhance their efficacy through complementary bioactivities. Raimo *et al*. engineered a composite preparation by co-isolating PDEVs from grapes, blood oranges, papayas, pomegranates, and oranges. In an H₂O₂-challenged oxidative-stress model, this mixed preparation of PDEVs fully restored mitochondrial membrane potential and suppressed the generation of mitochondrial superoxide [90]. These findings collectively establish PDEVs as potent antioxidants and anti-inflammatory agents suitable for wound therapy.

#### Proliferation and migration

Effective tissue regeneration requires the coordinated proliferation and migration of resident cells to repopulate the defect and restore tissue integrity [91]. Cellular proliferation facilitates rapid repopulation of the wound through accelerated cell cycle progression, which supports neoangiogenesis and the formation of granulation tissue. Concurrently, cellular migration from the wound margins contributes to defect closure through integrin-dependent adhesion dynamics, extracellular matrix (ECM) remodeling, and phagocytic clearance of necrotic debris [92].

MAPK and PI3K/Akt are among the key signaling pathways through which PDEVs regulate cell proliferation, migration, and tissue repair. The preferences of PDEVs derived from different sources show significant variations, which may be closely related to the composition of their bioactive molecules. PDEVs from *Morinda officinalis* activate the MAPK/YAP1 pathway, thereby promoting endothelial cell proliferation, migration [19]. In contrast, Gouqi-derived nanovesicles activate the PI3K/Akt/mTOR/p70S6K/4EBP1 cascade, thereby enhancing the proliferative activity of MC3T3-E1 pre-osteoblasts [70]. In addition, *Lycium ruthenicum* Murray-derived exosome-like nanovesicles inhibit Aβ-induced apoptosis in PC12 cells *via* the MAPK and PI3K/AKT signaling pathways, further suggesting that these pathways also participate in PDEV-mediated cell survival responses [67].

Furthermore, some PDEVs can also indirectly promote cell proliferation and migration by regulating the expression of ECM-related proteins. For instance, grapefruit-derived PDEVs enhance HaCat cell proliferation and migration by upregulating the expression of COL1A1 and fibronectin [53]. Similarly, PDEVs from wheat [93], *Solanum lycopersicum* [94], *Cissus quadrangularis* [95], and *Opuntia ficus-indica* Fruit [86] have demonstrated comparable pro-proliferative and pro-migratory activities. It should be noted that the specific molecular mechanisms underlying the functions of these PDEVs remain partially elucidated. While most studies have preliminarily identified downstream effector molecules or signaling pathways, the primary active components, such as proteins, nucleic acids, and lipids facilitated by PDEVs and their transport mechanisms, still require further investigation.

#### Angiogenesis

Angiogenesis plays a pivotal role in wound healing. When a wound occurs, the disruption of vasculature and blockage of microcirculation lead to an ischemic and hypoxic microenvironment that initiates coagulation and inflammation. In chronic wounds, persistent inflammation continually harms vascular endothelial integrity and disrupts pro-angiogenic mechanisms. The newly formed blood vessels are essential as they deliver oxygen and nutrients required to sustain aerobic metabolism and enhance cellular viability at the wound site.

PDEVs have been reported to promote angiogenesis by enhancing cell proliferation, migration, and tube formation through source-dependent signaling mechanisms, including MAPK/YAP1, Akt/eNOS, and VEGF-related pathways [74, 75]. For instance, Zhao *et al*. confirmed that PDEVs derived from *Morinda officinalis* enhance endothelial cell proliferation, migration, and tube formation through activation of the MAPK/MEK1/2 pathway and upregulation of YAP1 and HIF-1α [19]. Notably, YAP1 serves as an integrator of mechanical and growth signals, potentially enhancing the sensitivity of pathways such as PI3K/AKT, while HIF-1α acts as a key transcriptional regulator of genes including VEGF, highlighting the functional connections between these signaling pathways. Similarly, Huo *et al*. demonstrated that PDEVs derived from *Salvia miltiorrhiza* root promote vascular endothelial angiogenesis by upregulating HMOX1 and activating the PI3K/AKT/MAPK pathway [73]. This finding suggests that PDEV treatment enables antioxidant responses and pro-proliferative signals to synergistically promote angiogenesis. Additionally, Tu *et al*. reported that PDEVs from *Dendrobium officinale* activate the Akt/eNOS pathway, upregulating eNOS and VEGFR2 [71], thereby fostering crosstalk between NO and VEGF signaling. Moreover, Cai *et al*. showed that PDEVs from *Epimedium brevicornu* Maxim. promote angiogenesis by activating the VEGF signaling pathway [72]. Collectively, these studies indicate that PDEVs do not merely activate multiple independent pathways but establish an integrated and mutually reinforcing signaling network through upstream integration and downstream convergence. This multi-target, self-synergistic mode of action could be crucial to their effective pro-angiogenic action in complex pathological environments.

#### Antibacterial activity

Wound infection poses a significant clinical challenge and a critical global public health issue, exacerbated by the mounting crisis of antimicrobial resistance. Wounds can become infected by a variety of pathogenic microorganisms, such as *Staphylococcus aureus* (*S. aureus*), *Pseudomonas aeruginosa* (*P. aeruginosa*), *Escherichia coli* (*E. coli*), *Candida albicans* (*C. albicans*), and various viral pathogens. The rapid evolution of multi-drug resistant strains and their capacity to develop robust biofilms often render standard antibiotic treatments ineffective. Infected wounds typically exhibit cardinal signs of inflammation, including increased pain, erythema, and localized warmth. In extreme cases, these infections may escalate to life-threatening conditions such as cellulitis, sepsis, or necrotizing fasciitis, which can potentially lead to mortality [96, 97].

PDEVs retain the biological activities of their parent plants due to their bioactive cargo. Researchers are currently exploring a diverse range of botanical sources for their potential in wound antimicrobial therapies. Dandelion (*Taraxacum mongolicum*), known in traditional Chinese medicine for its heat-clearing properties [98], produces extracts that exhibit broad-spectrum antibacterial activity [99]. Derived PDEVs from dandelion demonstrate antibacterial effects by binding to and neutralizing *S. aureus* exotoxins, significantly reducing their hemolytic activity [100].

Saroj *et al*. have extracted antibacterial PDEVs from mint. Their findings indicate that PDEV-loaded hydrogels result in significantly fewer viable colony-forming units of *S. aureus* and *E. coli* compared to controls after 3 hours of co-incubation. These mint-derived PDEVs disrupt the structural stability of bacterial cell membranes, leading to leakage of intracellular components and subsequent bacterial death [101]. Moreover, PDEVs from *Allium sativum* L and *Coptis chinensis* have shown potent antimicrobial properties [102, 103]. Mechanistically, these vesicles disrupt bacterial biofilms by removing the protective extracellular polymeric substances (EPS), thereby exposing the pathogens to host immune clearance. The variety of PDEV sources demonstrating validated antibacterial functions continues to grow. For example, vesicles derived from *Acanthopanax senticosus* not only exhibit direct antibacterial effects but also reprogram macrophages toward a pro-healing phenotype, facilitating synergistic wound healing [104]. In addition, ginger-derived nanovesicles, which are among the most thoroughly characterized PDEVs, release high concentrations of bioactive 6-gingerol and 6-shogaol. These compounds collectively inhibit both Gram-positive and Gram-negative bacteria, as well as fungal pathogens [105]. Furthermore, vesicles derived from *Houttuynia cordata* Thunb incorporated into collagen hydrogels have been shown to significantly reduce bacterial load and accelerate re-epithelialization in preclinical diabetic wound models [106]. These developments significantly enhance the phytochemical and functional diversity of PDEVs, making them promising agents for next-generation antimicrobial therapeutics.

In general, PDEVs exert their antimicrobial effects through a combination of direct bactericidal actions and host-mediated immunomodulation, although the relative contribution of each varies depending on the plant source and the target pathogen. The lipid bilayers of PDEVs can fuse with or insert into bacterial membranes, a mechanism particularly effective against Gram-positive species with exposed peptidoglycan layers, resulting in increased membrane permeability, membrane potential collapse, and leakage of intracellular contents. Concurrently, PDEVs serve as natural nanocarriers that concentrate and deliver bioactive phytochemicals, such as flavonoids, terpenoids, and alkaloids, at effective localized concentrations. These metabolites further disrupt membrane integrity, inhibit deoxyribonucleic acid (DNA) gyrase and topoisomerase activity, and interfere with bacterial energy metabolism. However, comprehensive mechanistic understanding of PDEV interactions with bacteria remains limited, prompting the need for further research in this field.

Despite showing promising antibacterial outcomes, the mechanistic understanding at the pathway level of PDEV-mediated antimicrobial activity lacks the development observed in studies focused on antioxidant (Nrf2-ARE) and anti-inflammatory (NF-κB) effects. Current evidence supports at least four mechanistic layers that operate in concert:

Direct membrane disruption: PDEV lipid bilayers, enriched in PA and specific phytosterols, can fuse with or insert into bacterial membranes. This mechanism is particularly effective against Gram-positive bacteria, which have exposed peptidoglycan layers, leading to increased membrane permeability, dissipation of the proton motive force, membrane potential collapse, and leakage of intracellular contents.

Bioactive cargo-mediated bactericidal action: PDEVs concentrate and deliver phytochemicals, including flavonoids, terpenoids, alkaloids, and phenolic acids, at locally effective concentrations. These metabolites are capable of inhibiting bacterial DNA gyrase and topoisomerase IV, disrupting cell wall synthesis, interfering with quorum sensing, and impairing bacterial energy metabolism by targeting the electron transport chain.

Anti-biofilm activity: garlic-derived and *Coptis chinensis*-derived PDEVs have been demonstrated to dismantle mature bacterial biofilms by degrading the EPS matrix. This process exposes sessile bacteria to the combined effects of both PDEV cargo and host immune attack. However, the precise molecular targets within the EPS, whether structural polysaccharides, eDNA, or biofilm-associated proteins, remain unidentified.

Host-mediated immunomodulatory antimicrobial enhancement: PDEVs indirectly augment antimicrobial defenses by promoting macrophage phagocytosis, enhancing neutrophil bactericidal capacity, and shifting the immune microenvironment from a chronic inflammatory state to a resolving state, thus restoring effective pathogen clearance.

Critically, pathway-level validation, comparable to the Nrf2-ARE or NF-κB axes, is presently lacking for antibacterial PDEVs. Future studies should employ methods such as transcriptomic and proteomic profiling of bacteria exposed to PDEVs, bacterial membrane lipidomics, quorum-sensing reporter assays, and systematic testing of minimum inhibitory concentration and minimum bactericidal concentration across standardized pathogen panels. Establishing defined molecular targets and dose–response relationships will be essential for positioning PDEVs as reliable alternatives or adjuncts to conventional antibiotics in wound care management.

#### Immunoregulation

Immunoregulation plays a critical role in wound healing processes. Following an injury, rapid immune activation fosters pathogen clearance and the removal of necrotic tissue, thereby cultivating a microenvironment conducive to tissue repair [107, 108]. A pivotal event is the polarization of macrophages from a pro-inflammatory (M1) to a reparative (M2) phenotype, a transition that is crucial for resolving inflammation and initiating proliferative remodeling. M2 macrophages produce growth factors (TGF-β and VEGF) and matrix regulators that enhance fibroblast proliferation, collagen deposition, and angiogenesis. In the context of chronic diabetic wounds, an impaired transition from M1 to M2 macrophages prolongs the inflammatory phase and significantly impedes tissue regeneration [109]. Additionally, a balanced immune response prevents excessive inflammation and associated tissue damage while also modulating scar formation through the regulation of the TGF-β3/TGF-β1 ratio. Therefore, immunoregulation not only orchestrates the initial defensive response but also integrates reparative signals throughout the healing cascade, ensuring effective tissue restoration.

Recently, Wu *et al*. demonstrated that *Pueraria lobata*-derived PDEVs facilitate M2 polarization by significantly downregulating pro-inflammatory genes (*IL-6*, *IL-1β*, *TNF-α*, *MCP-1*, *CD11c*) in M1 macrophages and upregulating anti-inflammatory genes (*IL-10*, *YM1*, *CD206*) in M2 macrophages [110]. This study confirms the substantial immunomodulatory capacity of PDEVs. Consistently, ginseng-derived exosomal miRNA has been shown to ameliorate rheumatoid arthritis by targeting KRAS-MAPK signaling in synovial macrophages [68], while *Taraxacum mongolicum* Hand.-Mazz*.*-derived EVs alleviate mastitis by modulating the NLRP3 inflammasome and NF-κB/MAPK pathways [69]. These findings suggest that PDEVs may regulate inflammatory responses through source-dependent molecular cargos and signaling pathways.

Despite their favorable immunomodulatory profiles, the translational potential of PDEVs is limited by significant knowledge gaps. Apart from influencing macrophages, the effects of PDEVs on other immune cell subsets, including neutrophils and T cells, which are critical in the healing of diabetic wounds, remain poorly understood [111, 112]. Future research should focus on these populations to propel the field forward. For instance, in chronic diabetic wounds, hyperactive neutrophils undergo excessive NETosis, resulting in the formation of neutrophil extracellular traps that exacerbate tissue damage and hinder epithelialization [113]. Investigating whether specific PDEVs can inhibit aberrant NETosis or enhance neutrophil efferocytosis to hasten the resolution of inflammation is vital. Moreover, the imbalance between pro-inflammatory T helper 17 (Th17) cells and immunosuppressive regulatory T cells (Tregs) characterizes non-healing wounds [112]. Research delineating whether PDEVs can influence T cell fate, for instance, by inducing Treg differentiation or curbing Th1/Th17 polarization, would represent a significant advance in reprogramming the systemic immune microenvironment.

Collectively, these immunoregulatory challenges, spanning both innate (neutrophils) and adaptive (T cells) immunity, underscore the need for systematic immunophenotyping studies using standardized PDEV preparations to elucidate source-specific immunomodulatory profiles.

### Separation of PDEVs

**Figure 3:**
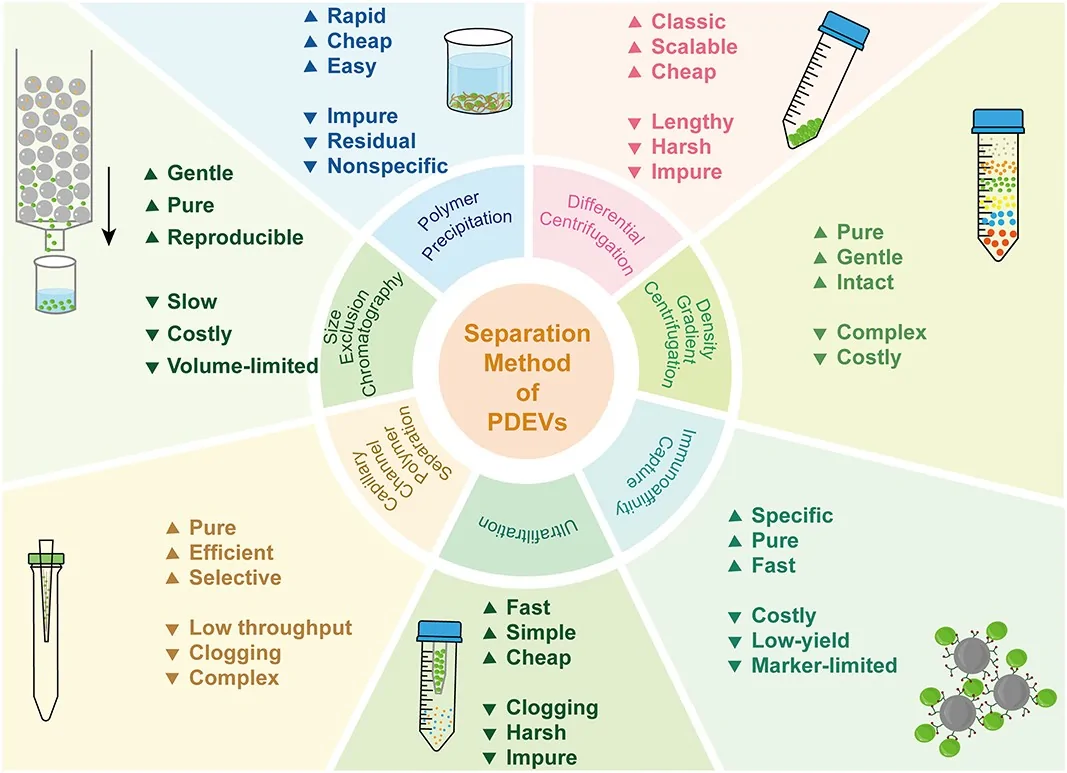
Common methods for separating plant-derived extracellular vesicles (PDEVs). These methods for separating PDEVs include polymer precipitation, differential centrifugation, density gradient centrifugation, immunoaffinity capture, ultrafiltration, capillary channel polymer separation, and size exclusion chromatography. These common methods exhibit distinct advantages and disadvantages for PDEV isolation and downstream applications

PDEVs exhibit diverse bioactivities intrinsically linked to their isolation methods. The selection of a separation technique significantly influences the yield, purity, and structural integrity of the isolated vesicles, which in turn affects their biological functionality and therapeutic potential. Common methods like differential centrifugation (DC), ultrafiltration, and polymer-based precipitation are extensively used for PDEV isolation (Figure 3).

#### Differential centrifugation

DC is the predominant method for isolating PDEVs, capitalizing on the unique sedimentation velocities of particles within plant juice. This process entails sequential application of low-, medium-, and high-speed centrifugation steps designed to remove large particles, dead cells, viscous proteins, fibers, and cellular debris. Subsequent ultracentrifugation, exceeding 100 000 × *g*, concentrates the PDEVs, which are then resuspended and stored at −80°C for future use [114, 115].

DC is advantageous due to its operational simplicity and efficiency in separation. Recent studies have successfully isolated PDEVs from various plants, including *Rhizoma Drynariae* [56], yam [116], carrot [117], *Herba Epimedium* [118], *Rabdosia rubescens* [119], *Pueraria lobata* [120], and ginseng root [121], which are noted for their significant morphological characteristics and high yield. Nonetheless, the primary limitations of DC include the relatively low purity of the isolated PDEVs, prolonged processing times, and reliance on specialized equipment. Quantitatively, while DC achieves high particle yields, the purity often falls within a moderate range. Studies have suggested that excessive centrifugation speeds might compromise the integrity of PDEVs, leading to an increase in soluble protein contamination.

#### Density gradient centrifugation

Density gradient centrifugation [122, 123] isolates PDEVs by resolving particles in plant juice based on buoyant density using media such as sucrose [124] or iodixanol [125]. This method segregates PDEVs into discrete layers and offers substantially higher purity compared to DC, making it ideal for applications that demand high-purity EVs.

Typically, density gradient centrifugation is employed alongside DC. For example, Tang *et al*. initially derived a crude vesicle suspension from rhubarb using DC, which was subsequently purified on a multi-step sucrose gradient ranging from 8% to 60%; PDEVs of varying sizes were then collected from specific interphases following ultracentrifugation [126]. Additionally, high-purity PDEVs derived from such sources as scallion [57], ginger [127], ginseng [128], and *Portulaca oleracea* L. [129] have also been isolated using this technique. Quantitatively, density gradient centrifugation can increase the particle-to-protein ratio to levels exceeding 10^10^ particles/μg protein by effectively removing non-vesicular aggregates. However, this centrifugation method is complex and can result in substantial vesicle losses during multiple washing and transfer steps, potentially affecting the overall efficiency and yield of PDEVs.

#### Polymer precipitation

Polymer precipitation is a commonly employed method for isolating PDEVs [130]. This technique utilizes hydrophilic polymers such as polyethylene glycol (PEG) to aggregate vesicles by disrupting their hydration layers and charge screening, leading to colloidal destabilization. Studies have demonstrated the effectiveness of this method by successfully precipitating PDEVs from tea leaf juice using 16% PEG6000 incubated at 4°C [131], and from ginger rhizomes using various types and concentrations of PEG (8%–15%) at the same temperature [132]. The method’s broad applicability is further evidenced by its successful scaling to isolate PDEVs from 14 different edible plants using 8% PEG8000 [133].

Although polymer precipitation offers advantages such as procedural simplicity, cost-effectiveness, and high yields compared to other vesicle isolation techniques, it has a significant drawback: compromised purity due to the persistent presence of polymeric residues [134, 135]. This inherent impurity typically limits its use in studies requiring high-purity PDEVs, especially in mechanistic analyses of vesicular functions. Thus, PDEVs obtained through polymer precipitation are recommended to undergo further purification steps.

#### Size exclusion chromatography

Size exclusion chromatography (SEC) separates PDEVs based on their specific migration paths and velocities within the gel column. This method typically employs stationary phases such as cross-linked dextran, agarose, and polyacrylamide [114, 136, 137]. In a recent study by Li *et al*., they removed crude fiber, large particles, and other impurities from the juice of *Artemisia argyi* leaves. The clarified supernatant was then processed through 200 nm and 750 kDa AKTA Flux columns (Cytiva, Logan, UT, USA), collected in an ice bath, and finally filtered through a 0.22 μm membrane to procure PDEVs [138]. Compared to alternative isolation methods, SEC not only produces vesicles with superior purity but also ensures a homogeneous size distribution. This technique is known for its high batch-to-batch consistency and the preservation of vesicular structural and functional integrity, thereby enhancing experimental reliability [139]. However, SEC faces challenges such as limited sample throughput, prolonged processing times, and potential matrix leaching.

#### Ultrafiltration

Ultrafiltration isolates PDEVs using microporous membranes with specific molecular weight cut-offs. This straightforward method avoids the complexities of extensive centrifugation and is amenable to large-scale processing. Despite its advantages, the potential for purity issues often necessitates its combination with other methods [140, 141]. For example, You *et al*. successfully isolated cabbage PDEVs using a sequence of PEG precipitation, ultracentrifugation, centrifugal ultrafiltration (100 kDa MWCO), and SEC [142]. Similarly, Steć *et al*. efficiently extracted EVs from citrus lemons by integrating ultrafiltration (100 kDa MWCO) with SEC [143]. These studies highlight the critical role of SEC in achieving high-purity PDEVs.

#### Capillary channel polymer separation

Capillary channel polymer (C-CP) separation represents an innovative technique that employs microfluidic-based polymer matrices to sieve and isolate PDEVs through microscale channels [144]. This approach, well-suited for high-throughput EV separation, was utilized by Jackson *et al*. to recover PDEVs from 20 types of fruits and vegetables, including ginger, cilantro, carrot, and strawberry. The process involves concentrating vesicles using the hydrophobic properties of C-CP fibers during centrifugation, followed by elution [145]. Despite its innovative nature, C-CP separation exhibits a low recovery rate and, while effective at the laboratory scale, continues to face challenges related to scalability and standardization for industrial applications.

#### Immunoaffinity capture method

The immunoaffinity capture method isolates PDEVs through precise antibody–antigen interactions, leveraging antibodies that target specific surface protein markers to ensure efficient and selective purification [146]. A typical tool in this method includes immunomagnetic beads. For instance, He *et al*. used Protein A-coated magnetic beads conjugated with a TET8-specific antibody to isolate TET8-positive Arabidopsis-derived vesicles [147]. Nevertheless, the immunoaffinity capture method encounters limitations such as cross-reactivity and limited binding affinity, which frequently necessitate the employment of expensive, highly specific antibodies that possess robust binding strengths.

### Identification of PDEVs

After isolating PDEVs, comprehensive characterization is crucial to confirm vesicular identity and purity [148]. Standard analytical parameters include morphology, particle size distribution (Table 2), zeta potential, and biomolecular composition [149, 150]. These metrics are essential for confirming vesicle integrity, stability, and functionality, which are critical quality attributes for their utilization in downstream applications and mechanistic studies.

**Table 2:** The extraction methods, particle sizes and zeta potentials of various PDEVs

EVs source	Isolation method	Particle size (nm)	Zeta potential (mV)	References
*Rhizoma drynariae*	DC	75.7 ± 15.8	−43.2 ± 0.04	[56]
Yam	DC	168 and 328	/	[116]
Carrot	DC	137.8	−16.5	[117]
*Herba Epimedium*	DC	120–140	/	[118]
*Rabdosia rubescens*	DC	151.4 ± 29.2	/	[119]
*Pueraria lobata*	DC	119–163	/	[120]
Ginseng	DC	142	/	[121]
Rhubarb	Density gradient centrifugation	165 and180	−18.7 and −29.19	[126]
Scallion	Density gradient centrifugation	121.2 ± 90.8	/	[57]
Ginger	Density gradient centrifugation	80.36	−15.92	[127]
Ginseng	Density gradient centrifugation	71.42	−18.8	[128]
*Portulaca oleracea* L*.*	Density gradient centrifugation	160	−31.4	[129]
Tea leaf	Polymer precipitation	274 ± 24.7	−20.6 ± 0.78	[131]
Ginger	Polymer precipitation	400 (with PEG)	−25	[132]
Blueberry, etc.	Polymer precipitation	189.62	−2.52	[133]
*Folium Artemisiae Argyi*	SEC	203	10.53	[138]
Cabbage	PEG Precipitation+Ultrafiltration+SEC	148.2 (PEG); 134.2 (Ultrafiltration); 98 (SEC)	−14.8 and −15.2	[142]
*Citrus limon*	Ultrafiltration+SEC	65.3 ± 31.9	/	[143]
*Solanum lycopersicum*	DC	130	−19.6	[94]
Gouqi	DC	171	/	[70]
*Atractylodes macrocephala*	DC	90.61 ± 19.66	/	[148]
*Catharanthus roseus*	DC	75.51 ± 10.19	−21.8	[65]

*EV* extracellular vesicle, *PDEVs* plant-derived extracellular vesicles, *DC* differential centrifugation, *SEC* size exclusion chromatography, *PEG* polyethylene glycol

#### Morphology

Morphology serves as fundamental evidence for the successful extraction of PDEVs. Morphological assessments are conducted using TEM [88, 151], scanning electron microscopy (SEM) [152], and atomic force microscopy (AFM) [140]. TEM and SEM provide high-resolution ultrastructural characterizations of PDEVs, revealing nanoscale features such as spherical, discoidal, and cup-shaped morphologies. Additionally, energy-dispersive spectroscopy (EDS), often incorporated within SEM/TEM platforms, provides elemental compositions of the vesicles [153]. Conversely, AFM, executed in intermittent-contact mode, not only displays 3D morphology but also measures the adhesion and stiffness of individual EVs *via* force spectroscopy.

#### Particle size

Unlike electron microscopy, nanoparticle tracking analysis (NTA) and dynamic light scattering (DLS) provide quantitative profiles of size distribution [154–156]. NTA measures the Brownian motion of individual vesicles to calculate their hydrodynamic diameter using the Stokes-Einstein equation, offering number-weighted data for size and concentration, particularly suitable for monodisperse systems. Conversely, DLS estimates size distributions by examining the fluctuations in light scattering caused by particle diffusion. Typically, the hydrodynamic diameters of PDEVs range from ~30 to 200 nm [65, 148]. For example, DLS has recorded sizes of 130 nm for Tartary buckwheat EVs [157] and 45.36 nm for those from *Viburnum opulus* [158]. Meanwhile, NTA documented pomegranate EVs at 152.8 nm with a concentration of 1.1 × 10^9^ particles/mL [159]. Both techniques facilitate accurate quantification of PDEV size.

#### Zeta potential

Zeta potential (ζ), indicative of the charge at the slipping plane, is predictive of PDEVs’ stability and *in vivo* behavior. Typically negative, ζ values with an absolute magnitude of 30 mV or higher denote high kinetic stability, while readings within ±30 mV are indicative of a propensity for aggregation. For instance, EVs from *Physalis peruviana*, with a ζ-potential of −6.7 mV, remained stable for only 2 weeks at 4°C [160]. In contrast, those from yam bean, with a ζ-potential of −26.5 mV, maintained stability for a month under the same conditions [161]. Measuring the ζ-potential is thus crucial for assessing the surface properties and stability of PDEVs.

#### Component identification

PDEVs consist of four primary classes of biomolecules: lipids, proteins, nucleic acids, and bioactive small molecules [162–164], and their systematic identification is critical for understanding their structure–function relationships.

The lipid components of PDEVs include PA, phosphatidylethanolamine (PE), phosphatidylcholine (PC), ceramides, and triacylglycerols. Techniques such as liquid chromatography-tandem mass spectrometry (LC–MS/MS) and high-performance liquid chromatography (HPLC) permit the identification and detailed characterization of these components [165], enabling structural annotation and precise quantification of these lipids. For instance, Cho *et al*. reported the isolation of 188 lipids from root-derived and cell-culture-derived EVs from ginseng, which were predominantly triacylglycerols, PCs, and diacylglycerols, as revealed by LC–MS [166].

Proteins are also abundant in PDEVs. Several membrane-associated proteins, including PEN1, PEN3, and TET8, have been proposed as candidate markers for PDEVs, while cytosolic proteins such as chaperones, HSP70/HSP90, and GAPDH are frequently detected in PDEV proteomic datasets. Techniques for protein analysis include western blotting (WB), LC–MS, flow cytometry, and protein quantification assays such as the bicinchoninic acid (BCA) method. Using these methods, Zhang *et al*. identified 243 protein species in PDEVs isolated from lemons [167].

Nucleic acids constitute a significant molecular class within PDEVs, among which miRNAs are prominently identified and quantified using high-throughput small-RNA sequencing. These miRNAs serve as post-transcriptional regulators in target cells, modulating various physiological pathways. Tan *et al*. detected 87 miRNAs ranging from 18 to 25 nt in length in Korean ginseng vesicles, demonstrating their substantial potential in regulating angiogenesis and other cellular behaviors [75].

In contrast to EVs derived from animals, PDEVs contain a plethora of bioactive constituents from their parent plants. These components are routinely isolated and quantified using HPLC and GC–MS techniques. For instance, Iriawati *et al*. utilized GC–MS to identify a suite of antioxidant metabolites within *Carica papaya* L. fruit-derived vesicles, including maltitol, 4-H-pyran-4-1-2, 3-dihydro3, 5-dihydroxy-6-methyl, methyl5, 12-octadecenedienoate, and phenol [168]. The stoichiometry of these bioactive constituents within the EVs dictates their integrated regulatory capabilities, resulting in immunomodulatory, antimicrobial, and antioxidant activities.

In addition to conventional methods for identifying PDEVs, techniques based on artificial intelligence (AI) and machine learning have proven more adept at analyzing EVs [169, 170]. Wei *et al*. developed the SVAtlas platform, which offers AI tools for marker analysis of EVs. Its compatibility with cross-species data enables its application in the multi-omics analysis of PDEVs [171].

### Overview of biomaterials in wound healing

Biomaterials are engineered for native biocompatibility to facilitate tissue repair and interfacing with biological systems. Various biological materials offer distinct advantages in loading PDEVs (Table 3) [100, 101, 103, 104, 106, 172–174]. In wound management, these materials function through multifaceted mechanisms including providing a physical barrier against pathogens [175]; regulating exudates through hydrophilic-hydrophobic balance [176]; delivering biochemical cues to modulate cell proliferation and migration [177]; sequestering cytokines for immunomodulation [178]; releasing growth factors to induce angiogenesis [179]; and inhibiting TGF-β to prevent fibrosis [180]. These synergistic actions enhance the kinetics and quality of healing. Crucially, the biocompatibility, functional adaptability, and structural controllability of biomaterials align well with the physicochemical properties and biological functions of PDEVs, making them ideal carriers for vesicle-based therapeutics. However, various biomaterial platforms exhibit distinct advantages and limitations for PDEV integration, necessitating careful selection based on fabrication conditions, microenvironmental characteristics, and release kinetics.

**Table 3:** Comparison of major biomaterial platforms for PDEV delivery

Biomaterial platform	Key structural characteristics	Advantages	Disadvantages	Impact on PDEV delivery and activity	References
Hydrogels	Water-swollen 3D cross-linked polymeric networks	Excellent biocompatibility; mimics ECM; high moisture retention; responsive to stimuli	Weak mechanical strength in pure forms; potential burst release of cargo	High suitability. Aqueous environment perfectly preserves PDEV membrane integrity; allows tunable, on-demand release	[100, 101, 106, 172, 173]
Films	Thin, flexible, 2D planar structures	Strong physical barrier against pathogens; excellent mechanical flexibility	Poor management of heavy wound exudate; limited 3D loading space	Moderate suitability. Good for spatial anchoring, but harsh solvents/temperatures during fabrication risk denaturing PDEVs	[174]
Microneedles	Micro-scale array device	Painless application; physically bypasses the stratum corneum barrier	Limited loading capacity; complex fabrication techniques required	High efficiency. Delivers PDEVs directly into deep dermal layers without harsh pressure/temperature stress	[103, 104]
Aerogels	Ultra-lightweight solids with hierarchical porosity	Exceptional exudate absorption capacity; massive specific surface area	Brittle without reinforcement; synthesis requires extreme drying processes	Massive surface area for loading, but requires robust lyoprotectants to prevent freeze-drying–induced PDEV rupture	Prospective

*PDEV* plant-derived extracellular vesicles, *ECM* extracellular matrix

#### Hydrogel

Hydrogels are insoluble, water-swollen 3D networks that mimic the native ECM, offering significant biocompatibility and tunable properties. Their high exudate absorption capacity and efficacy in promoting cell adhesion and migration make them excellent candidates for wound healing applications [181, 182]. These networks are created through either physical or chemical crosslinking processes [183, 184].

Among various biomaterial platforms, hydrogels exhibit superior PDEV-loading capabilities due to the exceptional compatibility of their 3D cross-linked networks with the physicochemical characteristics of PDEVs. This structural superiority is supported by three key features: (i) highly hydrophilic, aqueous environments that preserve vesicle membrane integrity and bioactive cargo [172], (ii) tunable porosity ranging from 10 to 500 nm, which can be precisely adjusted through crosslinking density to align with the 30–300 nm dimensions of PDEVs, and (iii) mild crosslinking conditions that prevent thermal or chemical denaturation of the vesicular cargo. Moreover, by integrating pH-, enzyme-, or temperature-responsive mechanisms with adjustable biodegradability, smart hydrogels can ensure precise, demand-responsive release of PDEVs directly into the wound bed [106]. Additionally, the inherent tissue adhesiveness of these materials prolongs PDEV retention at the defect site, while their compliant texture and adjustable mechanical strength ensure optimal adaptation to dynamically moving skin [106, 173]. This combination of structural and functional specificity renders hydrogels an ideal choice for composite carriers of PDEVs.

Beyond traditional static networks, dynamic and multifunctional biomaterial paradigms substantially enhance PDEV delivery. Self-healing hydrogels, characterized by dynamic reversible bonds, can spontaneously repair damage induced by movement [185], potentially preventing dressing fractures and managing premature burst releases of PDEVs. Furthermore, Janus dressings, featuring asymmetric structures, include a hydrophobic outer layer to repel bacteria and a hydrophilic inner layer to promote tissue adhesion [186], facilitating targeted release of PDEVs into the wound bed. Additionally, conductive hydrogels that incorporate elements such as conductive polymers, MXenes, or liquid metals simulate the skin’s native electrical microenvironment [187]. By synergizing biophysical electrical cues with PDEV-mediated biochemical signaling, these electroactive materials may actively promote electrotaxis-driven cell migration and nerve regeneration, especially beneficial in treating diabetic ulcers.

Particularly for diabetic wounds, the persistently elevated glucose levels in the local microenvironment constitute a primary healing barrier. To address this metabolic challenge, hydrogels can be engineered into glucose-responsive smart platforms. For example, incorporating glucose-consuming enzymes like glucose oxidase (Gox) or glucose-responsive boronic acid derivatives into the hydrogel network can actively reduce excessive local glucose concentrations. These glucose-responsive smart hydrogels not only help normalize the metabolic microenvironment but also utilize glucose-triggered biochemical changes to control the on-demand release of encapsulated PDEVs, maximizing therapeutic efficacy.

**Figure 4:**
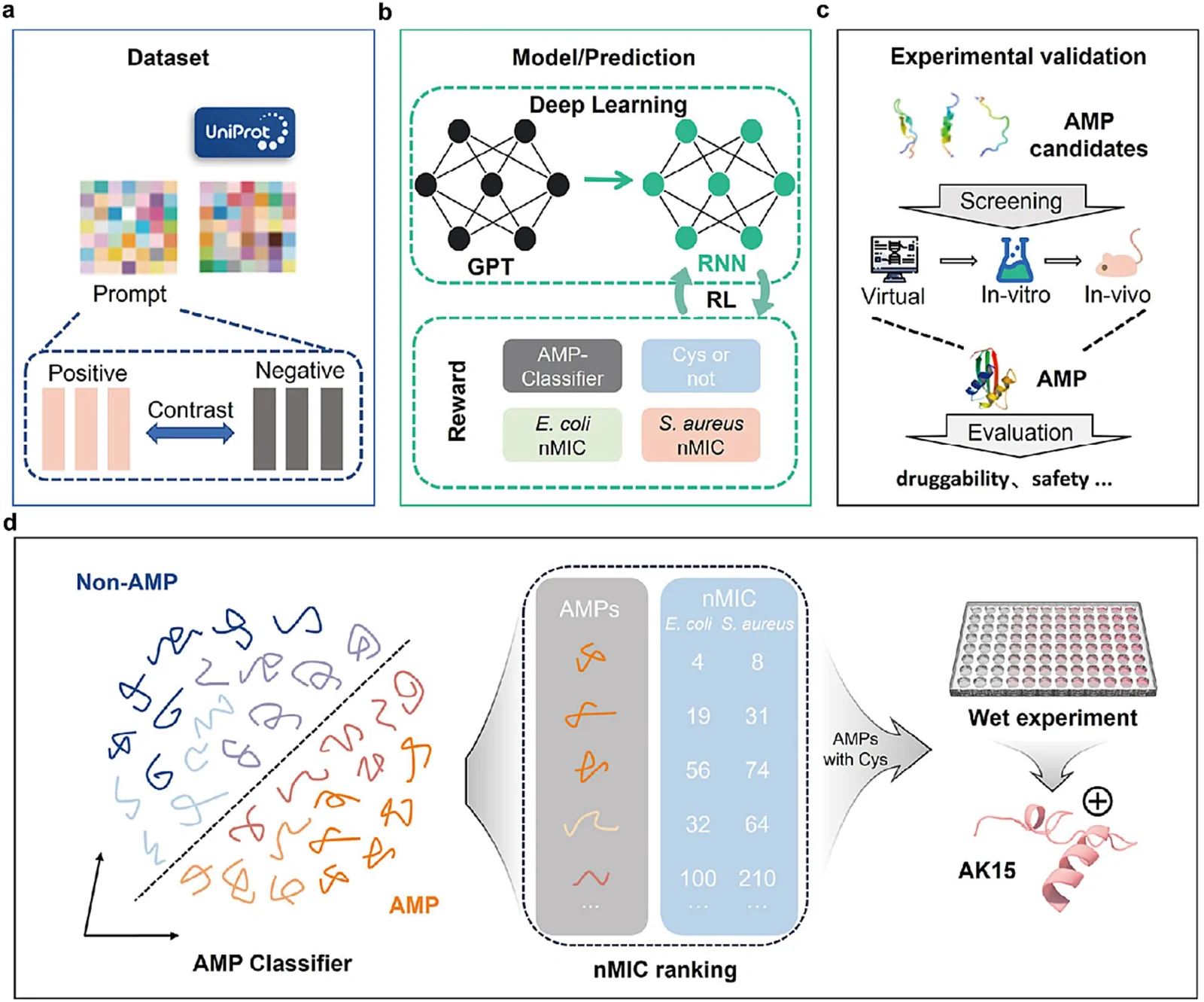
Generation and screening of AK15 using AMP-hydrogel-Designer. (**a**) Training dataset of AMP-hydrogel-Designer. (**b**) Architecture of AMP-hydrogel-Designer. (**c**) Experimental validation of AMP candidates. (**d**) Detailed generation and screening steps of AK15 [188]. Copyright 2025, Wiley. *S. aureus Staphylococcus aureus*, *E. coli Echerichia coli*

These structural and functional evolutions inherently increase the complexity of biomaterial design, steering the field toward data-driven methodologies. The capabilities and current limitations of AI in this area are critically evaluated in the subsection “AI-Driven Development Framework” within the Conclusion and Perspectives section. Recently, AI has shown considerable promise in enhancing hydrogel design and facilitating dynamic wound monitoring. For instance, Jiang *et al*. incorporated a generative AI model, AMP-hydrogel-Designer, with multi-objective optimization techniques to create a novel, broad-spectrum antimicrobial peptide named AK15. This peptide was then integrated with a PEG-4SH hydrogel and Cu-BTO to establish an intelligent, dynamically cross-linked network aimed at precise treatment of drug-resistant infections (Figure 4) [188]. Beyond structural innovation, AI also supports the monitoring of wound conditions. Yuan *et al*. employed a machine learning-based decision-making process along with regenerative bioelectronics to develop conductive hydrogel patches. This system tracks changes in wound conditions and dynamically optimizes treatment parameters *via* reinforcement learning techniques. Initially, during the infection stage, high current pulses (4 mA) are applied to swiftly release Ga^3+^ ions for broad-spectrum antibacterial effects; as the healing process continues, the AI controller gradually reduces the stimulus intensity (0–2 mA) to facilitate tissue regeneration [189].

However, these AI applications have clear limitations. The data used for training these systems are often narrow and specific, which restricts their generalizability to broader polymer systems. A more intrinsic limitation is that while algorithms excel in controlled environments, they struggle to replicate the complex, dynamic *in vivo* wound settings. Moreover, the AI-driven design of PDEV-biomaterial composites is further limited by the scarcity of data. Given that PDEVs vary naturally and lack standardized data on their molecular profiles and release kinetics, AI struggles to predict their interactions with biomaterials. Addressing this data gap is essential for any AI-based advancements in this field.

#### Microneedle

Microneedles (MNs) are a type of device consisting of micrometer-scale arrays, with individual needles typically measuring 10–1000 μm in length and 1–100 μm in diameter. Their primary function is to create minute channels in skin or wound tissues *via* physical puncturing, without harming dermal blood vessels and nerves [190, 191]. During the wound healing process, MNs penetrate the biological barrier of the wound through minimally invasive channels, enabling deeper tissue penetration for drugs and active agents, thus circumventing the issue of low bioavailability presented by the skin’s stratum corneum when using traditional topical applications [192, 193].

MNs can effectively deliver PDEVs directly to the dermal layer of skin *via* micron-sized channels, avoiding obstructions by the stratum corneum or loss through exudation. This method significantly enhances bioavailability compared to traditional topical applications. Additionally, the minimally invasive delivery process is gentle, eliminating the need for harsh conditions such as high pressure or temperature, thus preserving the structural integrity of the PDEVs membrane and the bioactivity of its cargo.

Li *et al*. developed a transdermal MN system that utilizes nanovesicles derived from garlic, combined with immunomodulatory garlic polysaccharides. The design of the MN facilitates both rapid and controllable release of the garlic vesicles [194]. Conversely, Long *et al*. encapsulated PDEVs derived from *Portulaca oleracea* L*.* within hyaluronic acid-based MNs. By using a soluble MN formulation, they markedly enhanced the therapeutic efficacy of these PDEVs [66]. These studies demonstrate the suitability of MNs as a platform for loading PDEVs. By adjusting MN parameters such as length, stiffness, and PDEV loading according to the type of wound, treatment can be optimized for greater precision and efficiency.

#### Film

Films, thin and flexible biomaterials commonly made from polymers such as polyurethane or silicone, are ideal for wound coverage and protection due to their tailored properties [195, 196]. They can be prepared through various methods including vapor deposition, spin coating, and electrospinning [197, 198]. Among these methods, electrospun nanofiber membranes are notable for their high porosity, large specific surface area, and tunable fiber architecture, which enhance cell adhesion and proliferation and offer a straightforward fabrication process [199].

Films are highly effective in achieving directional enrichment and localized anchoring of PDEVs due to their dense and tunable two-dimensional planar structure. The integrated advantages of physical protection and structural support are considerable: films encapsulate PDEVs through appropriately sized pores and also provide mechanical protection to wounds with their adjustable mechanical properties, thereby preventing the loss of PDEVs in dynamic environments. However, it is crucial to acknowledge that films and membranes typically necessitate high-temperature processing or the use of organic solvents during fabrication. These conditions may compromise the bioactivity of PDEVs and the integrity of the membrane. Pratiwi *et al*. verified that membranes composed of a cellulose acetate matrix can act as storage matrices for cranberry nanovesicles, thereby ensuring their long-term stability at 4°C [174]. This finding indicates that carefully optimized film fabrication protocols can mitigate processing-related damage, making films a viable option as composite carriers for PDEVs under biocompatible preparation conditions.

#### Prospective carriers: aerogels

While hydrogels and films are currently established platforms for PDEV delivery, aerogels represent a highly promising, though critically unexplored, frontier. Aerogels are ultralight, biocompatible solids characterized by hierarchical porosity, high surface area, and tunable pore volume, making them ideal for advanced wound care. They absorb excess fluid effectively through capillary action while maintaining optimal moisture levels, which accelerates re-epithelialization and overall wound healing [200, 201].

Although no published research has explicitly demonstrated the loading of PDEVs onto aerogels, their unique structural properties offer theoretical advantages for future composite design. The 3D interconnected pores in aerogels allow efficient exchange of nutrients and metabolites, while their robust physical barrier properties effectively block enzymatic erosion, potentially extending the functional duration of PDEVs. Furthermore, successful precedents in mammalian systems justify exploring this direction; for instance, platelet-derived EVs have been successfully loaded onto biosynthetic cellulose aerogels, confirming the suitability of the aerogel structure for EV applications [202].

However, translating this theoretical potential into practical applications poses specific technical challenges. Aerogel synthesis involves processes such as ambient pressure drying, supercritical drying, and freeze-drying [203–205]. For integrating PDEVs, the freeze-drying fabrication method poses significant dehydration stress risks to vesicle membranes, potentially compromising the structural integrity and bioactivity of the PDEVs. Consequently, it is anticipated that PDEV-aerogel composites will become a major research focus. Realizing this potential, however, relies on overcoming two technical challenges: developing effective lyoprotectants and refining drying protocols that preserve vesicle integrity.

In summary, among the biomaterial platforms discussed, hydrogels have the strongest experimental evidence supporting PDEV integration. Films and MNs provide emerging yet growing support. Aerogels, while structurally appealing, remain at the conceptual stage for PDEV delivery and require rigorous experimental validation before they can be considered alongside established platforms.

### Comparative analysis and design principles for PDEV–biomaterial composites

The selection of suitable biomaterial platforms for the integration of PDEVs necessitates a systematic approach to evaluating material-vesicle interactions. Hydrogels are identified as the optimal choice owing to their aqueous microenvironment, adjustable porosity, and gentle fabrication conditions, which together help to maintain the integrity of PDEV membranes and the bioactivity of their cargo. MNs demonstrate superior efficiency in transdermal delivery but their application is restricted to certain types of wounds that require deep tissue penetration. Films provide exceptional wound coverage and mechanical protection, although their fabrication conditions must be meticulously managed to prevent thermal or chemical damage to the PDEVs. Aerogels are outstanding in managing exudate but require specialized processing to prevent dehydration-induced damage to the vesicles. Nanoparticle carriers facilitate targeted delivery but require surface conjugation chemistry that can alter the surface proteins and recognition properties of PDEVs.

Consequently, when designing PDEV-biomaterial composites, researchers should adhere to four fundamental principles: (i) biocompatible fabrication conditions that protect vesicle integrity; (ii) hydrophilic microenvironments that ensure membrane stability; (iii) adjustable pore structures that accommodate the dimensions of PDEVs while facilitating controlled release; and (iv) degradation kinetics that are synchronized with wound healing timelines. These principles establish a logical framework for optimizing the performance of PDEV-biomaterial composites in various wound healing applications.

### Advances in PDEV—biomaterial composites

#### Advantages of composite material

*PDEV stabilization.* PDEVs possess inherently unstable membrane structures. Their phospholipid bilayers, rich in unsaturated fatty acids and substantially deficient in cholesterol and sphingolipids, exhibit excessive membrane fluidity and inadequate mechanical strength, making PDEVs highly vulnerable to environmental variations in temperature, pH, and ionic strength, which can induce vesicle fusion or rupture [139]. Unlike animal-derived vesicles, the surface proteins of PDEV membranes, such as thylakoid membrane proteins and cell wall-associated proteins, primarily function in metabolic regulation and defense, rather than providing structural support. This structural limitation renders PDEVs less resistant to degradation by lipases and proteases.

In wound microenvironments, ROS and fluctuating pH levels further destabilize PDEVs, increasing their susceptibility to structural failure. Thus, enhancing the stability of PDEVs is vital for therapeutic efficacy. Biomaterials such as hydrogels and films, constructed from stable polymers, significantly reinforce vesicle integrity through physical protection of both the membrane structures and the molecular cargo. For instance, a study demonstrated that EVs derived from *Perilla frutescens* leaf, when encapsulated in hydrogels, maintained remarkable stability at 4°C, showing only a minimal increase in particle size from 155.7 nm to 162.8 nm over 30 days, whereas free PDEVs in solution exhibited significant aggregation, with an increase in diameter from 155.7 nm to 172.9 nm [206].

Vesicular cargo stability is significantly enhanced. For example, Liu *et al*. encapsulated curcumin within banana-derived EVs, which were integrated into a semi-interpenetrating network hydrogel. This hydrogel was composed of low-methoxyl pectin, sodium alginate, and crosslinked calcium ions. When exposed to UV irradiation, the curcumin in free vesicles exhibited only a 46.8% retention of activity after 24 hours, compared to 95.5% in those encapsulated within the hydrogel. This finding indicates that the hydrogel acts as a physical barrier that protects against photodegradation. Similarly, when subjected to elevated temperatures, curcumin in free vesicles showed a 50% retention of activity after 1 hour, whereas it maintained over 85% activity when encapsulated in the hydrogel. These results confirm that hydrogel integration significantly enhances the stability of PDEVs and preserves the activity of encapsulated curcumin [207].

*Controlled release.* PDEVs, which are rich in bioactive components, fulfill dual roles: they function as both direct therapeutic agents in wound repair and as delivery vehicles for small molecules such as polyphenols and flavonoids [208]. However, their application to wounds exposes them to rapid degradation by proteases and clearance by bodily fluids. Free PDEVs typically lose their therapeutic bioactivity within several days. Additionally, their clinical application is impeded by stringent −80°C storage requirements to prevent aggregation [209]. The integration of PDEVs with advanced biomaterials addresses these challenges by substantially enhancing stability. Through optimized matrices serving as physical shields, the *ex vivo* storage period of PDEVs can be extended at 4°C or even at room temperature [210].

The integration of PDEVs with biomaterials establishes a controlled-release system designed to (i) extend vesicle retention at the wound site, thereby prolonging the therapeutic effects; (ii) enhance the release and efficacy of small molecules, improving wound healing outcomes.

The release kinetics of PDEVs from biomaterial composites are influenced by the characteristics of the materials used. In the case of non-responsive biomaterials, release of PDEVs is predominantly governed by physical properties such as porosity and surface charge, resulting in a passive diffusion mechanism that typically provides stable vesicle release. For instance, Liu *et al*. loaded *Perilla frutescens* leaf-derived EVs into a Carbopol 940 hydrogel, achieving only a 55% cumulative release after 72 hours. Fluorescence imaging further demonstrated that vesicle signal intensity was twice as high in the skin after 6 hours for hydrogel-delivered vesicles compared to free vesicles, confirming sustained release and significantly prolonged retention at the lesion site [206]. Additionally, Zhong *et al*. incorporated turmeric-derived EVs into a hydrogel formed through coordination between hyaluronic acid-dopamine and Fe^3+^, stabilized by coordination bonds, hydrogen bonds, and π-π interactions. This configuration led to a cumulative vesicle release of 96.7% over 5 days [211]. Furthermore, Wang *et al*. used a calcium alginate hydrogel to load coriander-derived EVs, achieving sustained release over three days. This extended release period is crucial for maximizing the therapeutic potential of these vesicles in wound healing [212].

Stimuli-responsive biomaterials offer significant advantages over non-responsive systems for advanced applications. When integrated with PDEVs, these materials facilitate the on-demand release of PDEVs in response to specific endogenous stimuli at the wound site, such as ROS or elevated enzyme activity within inflamed regions. This targeted release mechanism enhances therapeutic precision. For example, pH-responsive systems utilize bonds such as imine or hydrazone linkages [213, 214], whereas ROS-responsive systems deploy bonds like boronated esters [215, 216].

Current research primarily focuses on animal-derived EVs loaded onto responsive biomaterials [217, 218], with limited exploration in the realm of PDEVs. Saroj *et al*. developed an oxidized alginate-chitosan hydrogel crosslinked *via* imine bonds, which was then loaded with peppermint-derived EVs. Under physiological conditions, the cumulative release of PDEVs reached ~10% on Day 1, 45% on Day 3, and plateaued at ~60% by Day 7 [101]. However, the lack of release data under acidic conditions raises questions about the hydrogel’s pH-responsive functionality. Nevertheless, the targeted release from biomaterials loaded with PDEVs appears more conducive to wound healing.

The integration of PDEVs into biomaterials governs both the vesicular release kinetics and the delivery of encapsulated small molecules. Structurally, PDEVs comprise a phospholipid bilayer membrane. This architecture includes hydrophobic fatty acyl domains and hydrophilic phospholipid regions, as well as an internal aqueous compartment, facilitating the co-loading of hydrophobic and hydrophilic therapeutics using techniques such as ultrasonication [219].

Notably, the pre-loading of therapeutics into vesicles prior to their integration into biomaterials contributes to controlled drug release. Yu *et al*. first encapsulated dimethyloxalylglycine (DMOG) in ginseng-derived EVs, then integrated these into a hydrogel. In contrast to the burst release seen with directly loaded DMOG (~40% within 24 hours), the PDEV-encapsulated DMOG exhibited a triphasic sustained release through the hydrogel (20% in the first 24 hours, reaching 50% by Day 6, and continuing through Day 14), thus maintaining therapeutic concentrations while minimizing systemic exposure [220].

In a similar vein, Wei *et al*. loaded oridonin into *Rabdosia rubescens*-derived EVs via extrusion before incorporating them into a semi-interpenetrating hydrogel. The EV-hydrogel system only achieved a 50% cumulative release of oridonin after 7 hours, in stark contrast to the direct-loading hydrogel, which released over 80% within 7 hours and peaked at 3.5 hours [119]. This controlled release is attributed to dual diffusion barriers: first through the vesicle membrane, then through the biomaterial matrix, which effectively prolongs therapeutic action and enhances efficacy.

Therefore, it is evident that biomaterials play a crucial role in the controlled release of active substances. In recent years, materials designed using machine learning have garnered research interest [221]. These materials not only possess enhanced controlled-release capabilities but also facilitate intelligent management and monitoring of the wound surface [222]. However, studies on vesicle-biomaterial composites in this context are currently limited.

**Figure 5:**
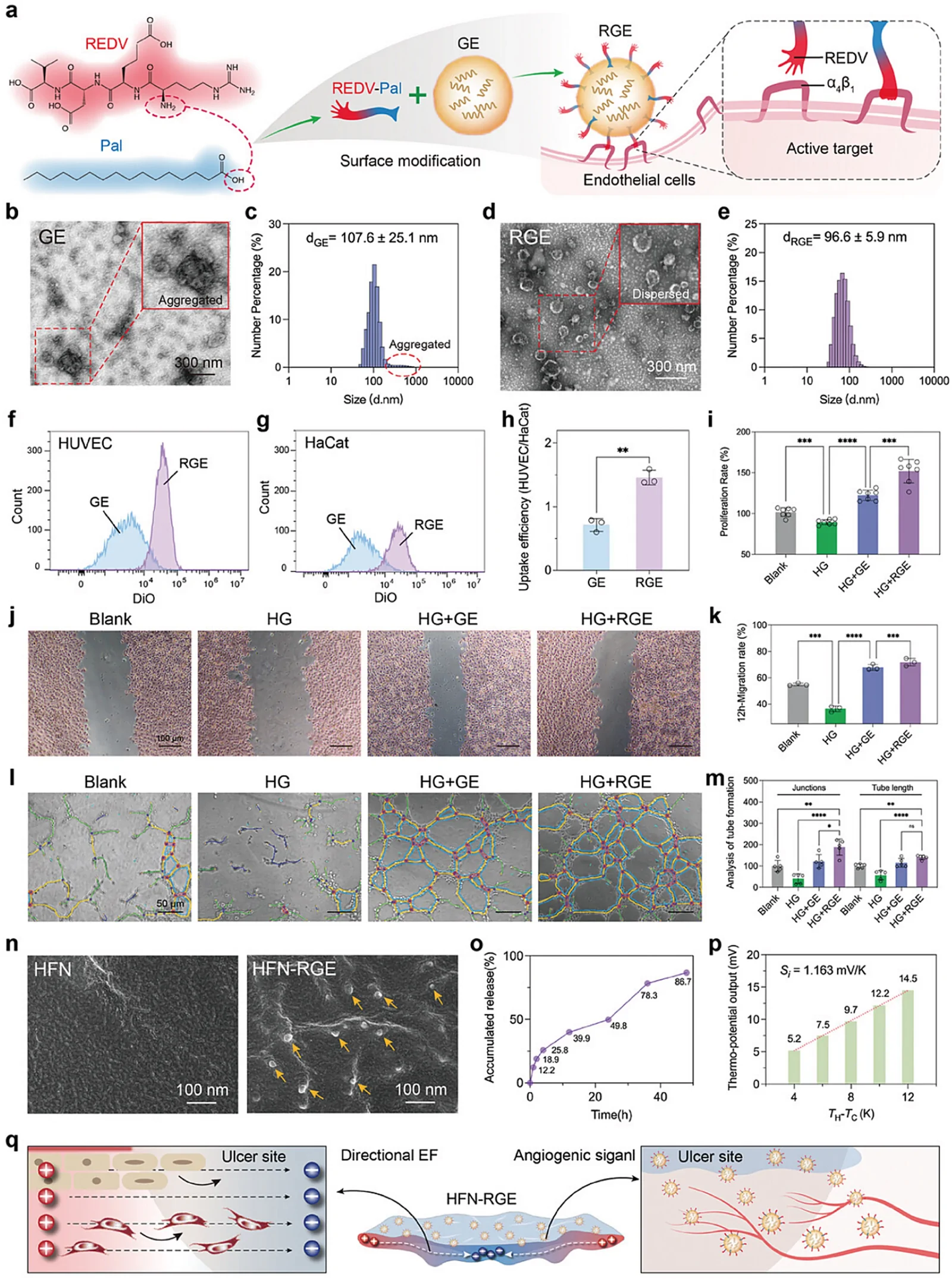
Characterization and evaluation of RGE and HFN-RGE. (**a**) Schematic diagram of the preparation of RGE. The RGE achieves specific targeting through selective recognition of the highly expressed α4β1 integrin on the surface of ECs. (**b–e**) TEM images and diameter distribution of GE (b and c) and RGE (d and e). (**f** and **g**) DiO-positive rates of HUVECs (f) and HaCats (g) co-cultured with the DiO-labeled GE or RGE for 12 h. (**h**) Quantitative analysis of HUVECs and HaCats cellular uptake efficiency. (**i**) HUVECs viability against blank, HG, HG + GE, or HG + RGE treatment. (**j** and **k**) Light microscope images (j) and quantified migration area (k) of HUVECs in different groups at 12 h. (**l** and **m**) Light microscope images (1) and quantified tube formation (m) of HUVECs on Matrigel in different groups at 9 h. (**n**) SEM images of HFN and HFN-RGE. The RGE was indicated by arrows. (**o**) RGE release profiles of HFN-RGE at 37°C within 48 h. (**p**) The thermoelectric test of HFN-RGE. (**q**) Schematic diagram of HFN-RGE providing directional EF concurrently supplying angiogenic exosomes [223]. Copyright 2025, Wiley. *RGE* REDV peptide-modified ginseng-derived exosomes, *HFN* HA and F127 Network, *EC* endothelial cells, *GE* ginseng-derived exosomes, *HUVECs* human umbilical vein endothelial cells, *HaCats* human immortalized keratinocytes, *EF e*ndogenous electric field, *TEM* transmission electron microscopy

*Synergistic effects*. PDEVs exhibit inherent therapeutic potency and a unique vesicular nanostructure. When engineered into biomaterial composites, they demonstrate enhanced bioactivity through dual mechanisms: (i) the sustained release of PDEVs prolongs retention at the wound site, maximizing antioxidant, anti-inflammatory, and pro-angiogenic functions; and (ii) the synergistic interaction between the biomaterial’s activity and progressively released PDEVs accelerates wound healing (Figure 5) [223]. For example, when dandelion vesicles are incorporated into hydrogel and applied to infected wound surfaces, they significantly accelerate the healing process. After three days, the wound healing rate was 36.12 ± 2.49%, compared to 25.19 ± 4.30% with hydrogel GelMA alone, and 21.55 ± 1.83% with only dandelion vesicles [100].

Similarly, Jin *et al*. incorporated lemon-derived EVs into a GelMA/DAS hydrogel, observing synergistic healing effects. In diabetic wound models, the EV-hydrogel composite promoted significantly faster closure compared to each component used individually. Over a 14-day healing period, the treatment attenuated inflammation, reduced wound width, and enhanced collagen deposition, indicating superior tissue remodeling [224].

Furthermore, the integration of PDEVs with various biomaterial carriers demonstrates extensive synergistic benefits. For instance, composites such as coriander EVs in GelMA hydrogels [212], mint leaf EVs in alginate-chitosan hydrogel [101], scallion EVs in dopamine-modified patches [57], and *Paris polyphylla* var. *yunnanensis*-derived EVs in PVP/CMC hydrogel [225] all facilitate sustained PDEVs release, maintaining effective therapeutic concentrations locally. Combined with the biomimetic properties of these carriers, these systems enhance angiogenesis, antioxidant/anti-inflammatory activity, and cell migration, consistently outperforming monotherapies. This illustrates the critical role of PDEV-biomaterial integration as a strategy for synergistic wound healing.

Beyond the scope of single-vesicle systems, the integration of multiple PDEVs into biomaterials significantly enhances wound repair efficacy. Miya *et al*. demonstrated this by co-incorporating *Aloe barbadensis*, *Azadirachta indica*, and *Zingiber officinale*-derived EVs within a PVA/chitosan nanomembrane, creating synergistic networks across different plant sources. This approach effectively targets multiple healing pathways simultaneously. In diabetic wounds, this multi-PDEV composite achieved a 93% closure rate, compared to 70% achieved by treatments using single vesicle types, highlighting the transformative benefit of combinatorial vesicle therapeutics [226].

*Engineered formats*. The integration of PDEVs with biomaterials significantly enhances their potential applications. Given the structural and compositional diversity of biomaterials, they can be processed into versatile composite systems. Utilizing adaptable fabrication techniques, engineers can create various forms such as hydrogels, aerogels, films, fibers, and nanoparticles. This morphological adaptability enables the development of tailored solutions that meet diverse research and clinical needs, thus facilitating broader biomedical implementations. Particularly with the rapid advancements in machine learning and the emergence of more predictive models [227, 228], these technologies are likely to promote more precise material construction. Concurrently, machine learning could offer more efficient tools for the preparation and analysis of PDEVs, including AI-driven frameworks for component identification and target prediction, which could accelerate the engineering of complex materials.

#### Preparation techniques for composite materials

Current fabrication strategies for PDEV-biomaterial composites are guided by the compatibility requirements between PDEVs and the biomaterial matrices. They primarily include three approaches: physical blending, physical adsorption, and chemical conjugation (Table 4).

**Table 4:** Various developed PDEV-biomaterial composites for wound healing

EVs source	Biomaterials	Loading techniques	Types of wounds	Healing effect	References
Turmeric	Metal-polyphenol hydrogel	Physical blending	Atopic dermatitis	The healing rate is >50% on the 7th day	[211]
Dandelion	GelMA hydrogel	Physical blending	Invasive wounds	The healing rate is 36.12 ± 2.49% on the 6th day	[100]
Scallion	SIS patches	Physical absorption	Wound in gastrointestinal anastomosis	Enhance CD31, VEGF, and reduce TNF-α	[57]
Coriander	Calcium alginate	Physical blending	Full-thickness incision	The healing rate is 96.21% on the 12th day	[212]
Mint leaf	Oxidized alginate-chitosan hydrogel	Physical blending	Infectious wounds	Complete healing within 10 days	[101]
Ginseng	Gallic acid-methacrylated gelatin hydrogel	Physical blending	Diabetic wounds	The healing rate is >90% on the 10th day	[220]
*Rabdosia rubescens*	Semi-IPN hydrogel	Physical blending	Wounds of chemoradiotherapy-induced oral mucositis	The healing rate is >80% on the 12th day	[119]
Lemon	Gelatin methacryloyl and dialdehyde starch hydrogel	Physical blending	Diabetic wounds	Complete healing within 14 days	[224]
*Paris polyphylla* var. *yunnanensis*	Polyvinyl pyrrolidone/carboxymethyl chitosan hydrogel	Physical blending	Full-thickness incision	The healing rate is 82.87 ± 4.77% on the 7th day	[225]
*Aloe barbadensis*, *Azadirachta indica*, and *Zingiber officinale*	Chitosan-based nanomembrane	Physical blending	Diabetic wounds	Complete healing within 14 days	[226]
*Houttuynia cordata* Thunb	Collagen hydrogel	Physical blending	Diabetic wounds	The healing rate is >90% on the 10th day	[106]
Cactus	Gelling sprayable hydrogels	Physical blending	Acute wounds; third-degree burn and scald composite full-thickness incision	Complete healing within 15 days	[173]
*Momordica charantia* L.	GelMA-dopamine hydrogel	Physical blending	Diabetic wounds	The healing rate is >95% on the 15th day	[229]

*SIS* small intestinal submucosa, *VEGF* vascular endothelial growth factor, *TNF-α* tumor necrosis factor alpha

**Figure 6:**
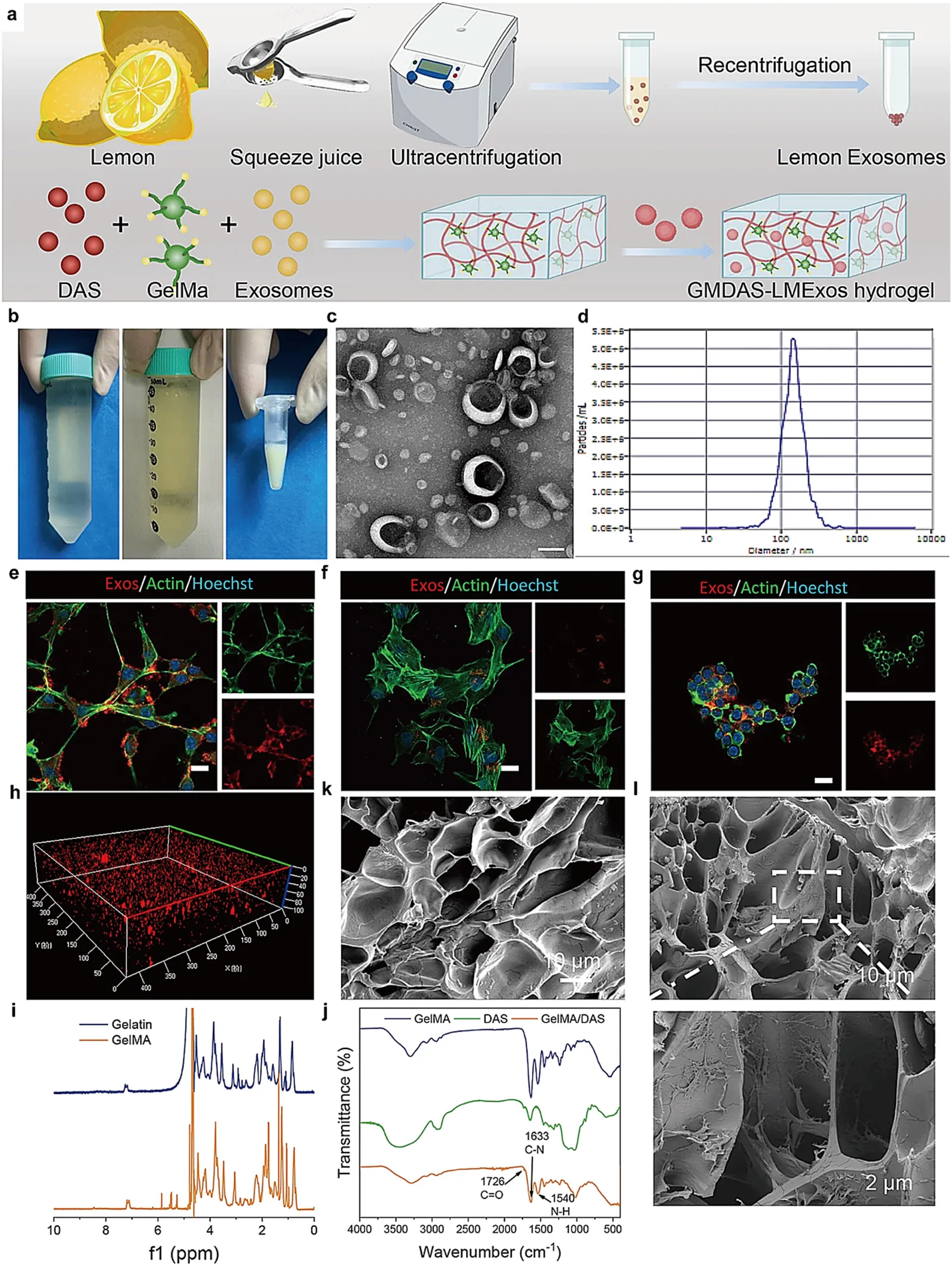
Preparation and characterization of GelMA/DAS/Exo hydrogel. (**a**) Diagram of the synthesis of hydrogels. (**b**) Physical picture of lemon exosomes. (**c**) Lemon exosome TEM. (**d**) Lemon exosome NTA. (**e–h**) Lemon exosomes could be targeted by macrophages, fibroblasts and HUVECs and could be uniformly loaded in a hydrogel. (**i**) ^1^H NMR spectra of gelatin and GelMA. (**j**) IR spectra of the GelMA, DAS, and GelMA/DAS hydrogels. (**k** and **l**) SEM images of the GelMA/DAS and GelMA/DAS/Exo hydrogels [224]. Reprinted under CC-BY license. *HUVECs* human umbilical vein endothelial cells, *GelMA/DAS/Exo* hydrogel Gelatin Methacryloyl/Dialdehyde starch/Lemon exosomes hydrogel, *TEM* transmission electron microscopy, *NTA* nanoparticle tracer analysis, *^1^H NMR* nuclear magnetic resonance spectroscopy, *DAS d*ialdehyde starch, *SEM* scanning electron microscopy

*Physical blending.* This technique involves incorporating PDEVs into biomaterial matrices through non-covalent interactions [229]. The process typically entails two main steps: the isolation and purification of PDEVs followed by their homogeneous dispersion into biomaterial precursors such as hydrogels (Figure 6) or thin films. By modulating solvent polarity and ionic strength, PDEVs can be uniformly distributed within the matrix *via* hydrogen bonding or electrostatic adsorption. For example, Zhong *et al*. isolated turmeric-derived vesicles (zeta potential = −15.3 mV) through ultracentrifugation and blended them with dopamine-grafted hyaluronic acid (HD) and Fe^3+^. In this system, Fe^3+^ crosslinks HD *via* catechol coordination, while HD forms a 3D network through hydrogen bonds and π-π stacking. The vesicles stably attach to phenolic hydroxyl groups on HD *via* hydrogen bonds and van der Waals forces, ensuring their effective integration [211].

Importantly, physical blending allows the incorporation of PDEVs not only into physically self-assembling biomaterials but also into chemically crosslinked systems. For instance, Tan *et al*. engineered a composite hydrogel by integrating dandelion-derived EVs into a methacrylated gelatin (GelMA) precursor solution, followed by ultraviolet-triggered chemical crosslinking. Here, the dandelion-derived vesicles become stably immobilized within the porous 3D GelMA network through non-covalent interactions, preserving their structural integrity and bioactivity [100].

Physical blending is a straightforward and cost-effective method to incorporate PDEVs into biomaterials. It maintains their structural integrity and bioactivity while allowing tunable loading and release. However, it encounters challenges in achieving uniform distribution of PDEVs at scale and may result in premature leakage due to weak interfacial interactions.

*Physical adsorption.* Physical adsorption plays a crucial role in attaching PDEVs to biomaterial surfaces *via* non-covalent bonds such as van der Waals forces and electrostatic interactions. These interactions maintain the vesicles’ structural integrity and functional activity. This adsorption process is influenced by the physicochemical characteristics of the substrate. A notable study by Peng *et al*. involved functionalizing decellularized small intestinal submucosa (SIS) with dopamine to produce an SIS-dopa patch. The adsorption of PDEVs was accomplished by immersing the patch in a scallion-derived EV suspension and maintaining constant agitation at room temperature for 4 hours. The binding mechanism relies on hydrogen bonding between polar groups on the PDEVs membrane, such as hydroxyl and carboxyl, and the catechol moieties in dopamine. This is supported by electrostatic interactions between the positively charged dopamine and the negatively charged phospholipid bilayer of the PDEVs under neutral pH conditions [57].

Despite its advantages, physical adsorption presents several limitations for integrating PDEVs. The low-affinity, non-covalent bonds render the PDEVs susceptible to detachment under physiological shear forces, while the loading capacity remains inferior to that achieved through blending or covalent conjugation. Most importantly, physically adsorbed PDEVs lack protection against enzymatic degradation and environmental denaturation, which compromises their integrity and bioactivity.

*Covalent conjugation.* Covalent conjugation effectively anchors PDEVs to biomaterial substrates by forming robust bonds, typically between carboxyl groups on the material surface and amino groups on the PDEV membranes. This method provides enhanced binding and interfacial stability compared to physical adsorption. Although conventional EDC/NHS coupling ensures strong immobilization, its dependence on aggressive reagents restricts its applicability in delicate biological contexts. To overcome this, recent advancements in biomaterials for wound healing preferentially use dynamic covalent chemistry. For example, Schiff base formation is a common approach wherein aldehyde-functionalized biopolymers react spontaneously with primary amines on the PDEV surface under mild physiological conditions, forming dynamic imine bonds [230]. These covalent bonds are stable under typical conditions but can break down in the mildly acidic environment of infected wounds, allowing for controlled, responsive release of PDEVs.

**Figure 7:**
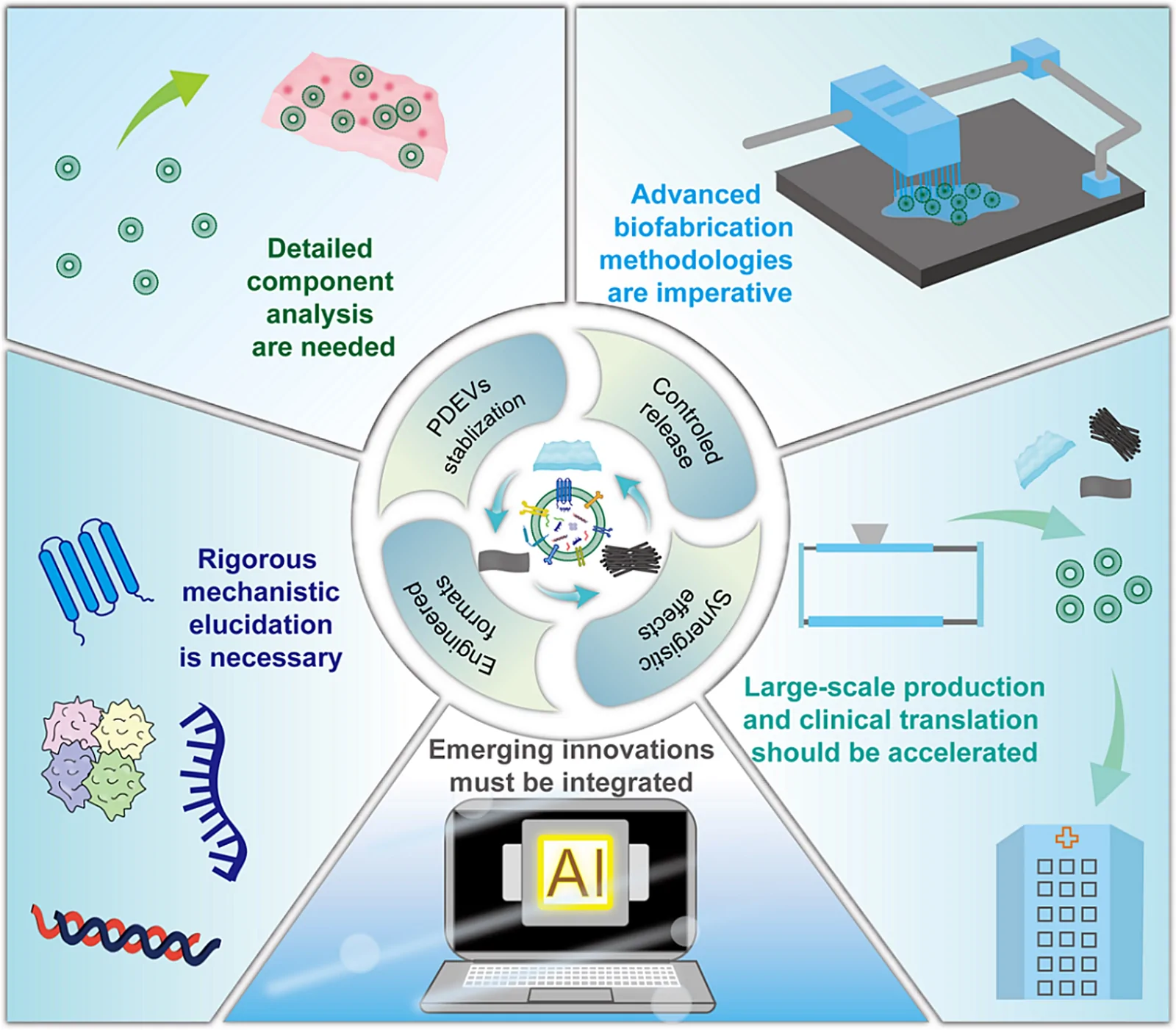
Plant-derived extracellular vesicle (PDEV)—biomedical composites and their future development directions. The future development directions for PDEV-biomedical composites include detailed component analysis, rigorous mechanistic elucidation, advanced biofabrication methodologies, large-scale production and clinical translation, and the integration of emerging innovations. These development directions are crucial for achieving PDEV stabilization, controlled release, synergistic effects, and optimized engineered formats.

Nevertheless, covalent conjugation methods come with their own drawbacks. Primarily, the use of external chemical reagents not only complicates the process but may also jeopardize the structural integrity and biological activity of the PDEVs. Thus, careful optimization of reaction conditions, such as pH, temperature, and reagent concentration, is crucial to mitigate functional loss while optimizing binding stability.

In summary, physical blending is superior for preserving PDEV bioactivity but provides limited control over release; physical adsorption offers moderate binding stability; and dynamic covalent conjugation excels in controlled release but necessitates meticulous optimization to minimize functional impairment. Consequently, the choice of fabrication strategy should be influenced by the specific requirements of the wound pathology, desired release kinetics, and permissible trade-offs in bioactivity.

*Surface modification strategies of PDEVs.* Beyond merely incorporating substances into biomaterials, the targeted engineering of PDEV surfaces has emerged as an essential methodological advancement to enhance their stability, specificity in cellular targeting, and integration into therapeutic scaffolds. Researchers have developed various strategies for such surface functionalization. One effective approach involves lipid insertion, where specific lipids, such as DSPE (1,2-distearoyl-sn-glycero-3-phosphoethanolamine), are anchored into the PDEV lipid bilayer through hydrophobic interactions. This anchoring facilitates the subsequent immobilization of aptamers *via* click chemistry [20]. Alternatively, covalent conjugation can directly link targeting molecules to PDEV surface proteins; however, this method may be hampered by slow reaction kinetics and pose risks of disrupting the vesicle surface’s structural integrity [231]. In contrast, non-covalent strategies that utilize electrostatic or hydrophobic interactions to attach ligands to the vesicle surface represent a promising, though relatively underexplored, approach [232]. Additionally, physical techniques, such as extrusion, sonication, and electroporation, can effectively alter the vesicles’ surface characteristics to facilitate the loading of functional molecules [233].

#### Design principles for composite materials

*Stability and biocompatibility.* The incorporation of PDEVs into biomaterial systems does not guarantee enhanced therapeutic outcomes, as efficacy critically hinges on overcoming several key integration challenges. Initially, physicochemical compatibility influences loading efficiency and distribution. Disparities in surface charge or hydrophilicity/hydrophobicity can result in irregular PDEV aggregation or adsorption. These mismatches may lead to the loss of bioactivity due to excessive binding or to ineffective delivery due to insufficient loading, both of which compromise therapeutic performance.

Furthermore, the processing conditions of biomaterials can jeopardize the integrity of PDEVs. High temperatures and cytotoxic crosslinkers may disrupt vesicle membranes and degrade bioactive cargo, while residual reactive groups may covalently modify functional proteins. Moreover, in metal-doped systems (Ag, Cu), excessive ion leaching may inactivate biomolecules by distorting enzymatic active sites.

Thus, a successful integration strategy should adhere to the following design principles: (i) engineer surface compatibility to enable efficient and controllable payload loading; (ii) adopt biocompatible processing methods that preserve vesicle integrity; and (iii) eliminate reactive components to maintain the functional integrity of the vesicles.

*Functional synergy.* PDEVs transport bioactive cargoes, such as polyphenols, flavonoids, and nucleic acids, which promote wound healing processes like cell proliferation and angiogenesis. However, their efficacy is limited by rapid degradation in the proteolytic wound microenvironment. While biomaterials provide a programmable platform with tunable properties, they often lack intrinsic bioactivity. Consequently, designing PDEV-biomaterial composites for functional synergy is crucial for enhancing therapeutic outcomes. Utilizing machine learning, it may be feasible to better predict the functional synergy of PDEV-biomaterial complexes, suggesting a significant future research direction.

### Challenges and future perspectives

Despite the demonstrated efficacy improvement through PDEV-biomaterial integration, significant challenges remain (Figure 7):

(1) **Inflammatory and allergic risks require rigorous safety profiling:** although PDEVs exhibit lower immunogenicity compared to animal-derived EVs and possess intrinsic anti-inflammatory properties, they still present certain allergenic risks. For instance, strawberry-derived EVs contain allergens such as Fra a 1 and Fra a 4 [234]. Clinical usage thus necessitates careful monitoring for allergic reactions. To enhance safety, detailed proteomic profiling of PDEVs is essential, along with the employment of purification techniques to remove allergenic components or modifications of vesicle surfaces to mask immune recognition epitopes. These strategies would facilitate the development of safer PDEVs for wound healing applications.
(2) **Biomaterial innovation and fabrication strategies remain basic:** current composites predominantly use simple hydrogel to preserve bioactivity. However, there is a significant unmet need for advanced functional biomaterials, particularly novel stimuli-responsive systems. Additionally, conventional techniques such as physical blending and chemical conjugation dominate composite synthesis, which severely restricts controlled-release kinetics and therapeutic efficacy. Advanced biofabrication methodologies, such as 3D bioprinting and microfluidic-assisted assembly, are crucial to address these limitations.
(3) **Insufficient mechanistic delineation impedes rational composite design:** the complex synergistic interactions, such as molecular recognition and dynamic biointerface regulation, as well as the specific properties of biomaterials affecting PDEVs, remain poorly understood. This knowledge gap not only hinders the identification of key regulatory nodes governing composite performance but also makes the rational optimization of composite design largely empirical. Key processes, such as tuning PDEV loading efficiency or modulating biomaterial microtopography for synergy, proceed without a predictable mechanistic framework to guide them.
(4) **AI-assisted development of PDEV-biomaterial systems: opportunities and limitations:** AI-assisted methods offer potential accelerations in the development of PDEV-bio-material therapies for wound healing by utilizing multidimensional datasets that encompass plant source selection, vesicle characterization, biomaterial formulation, release kinetics, and wound monitoring. Initially, machine learning models may correlate various parameters such as plant species, growth conditions, extraction parameters, omics profiles, and bioactivity assays with therapeutic outcomes to facilitate rational PDEV source screening. Subsequently, deep learning-based image analysis could enhance the reproducibility of PDEV characterization. This could be achieved by aiding in NTA, interpreting electron microscopy data, classifying vesicle morphology, and assessing batch quality. Furthermore, AI-guided optimization of formulations could enable predictions on how various factors, such as polymer concentration, crosslinking density, stiffness, degradation rate, pH sensitivity, ROS responsiveness, and enzyme-triggered cleavage, affect PDEV loading efficiency, stability, and release behavior. Additionally, simulations of release kinetics may assist in modeling PDEV delivery under specific wound conditions, which include exudate volume, pH, protease concentration, glucose levels, oxidative stress, and dressing deformation. Finally, AI can integrate into wound monitoring platforms to support personalized dressing changes and treatment modifications by analyzing wound size, color, temperature, exudate, infection risk, and healing trajectory.
  Despite these significant potentials, the utilization of AI in PDEV-biomaterial research is still nascent and encounters numerous challenges. The datasets currently available are often small, heterogeneous, and generated through non-standardized procedures for isolation, characterization, and potency assays. Consequently, models trained on such limited preclinical data are likely to be overfitted and might demonstrate poor generalizability. Moreover, AI-generated predictions necessitate experimental validations using standardized PDEV batches, clinically relevant wound models, and clearly defined biological endpoints. Challenges such as data interpretability, cross-center reproducibility, regulatory acceptance, and integration with good manufacturing practices (GMPs) are yet to be addressed. Therefore, at this stage, AI should be considered more as a decision-support tool rather than a replacement for mechanistic studies or rigorous preclinical validations.
(5) **Scalable production, standardization deficits, and clinical translation barriers:** the current production of PDEVs faces significant challenges including variability in plant batches, seasonal effects on plant growth, low yields, inadequate purity from conventional isolation methods, and a fluctuating supply of raw materials. These factors impede not only the medical applications but also the scalability of industrial manufacturing. A critical issue is the lack of standardized quality control frameworks, which poses a fundamental barrier to the clinical translation of PDEV-biomaterial composites. Despite encouraging preclinical outcomes, these composites have not progressed to clinical trials.
  For PDEVs to advance toward clinically translatable applications, standardized quality attributes should be established, including particle size distribution, morphological integrity, particle concentration, protein and lipid content, purity from non-vesicular contaminants, and source-specific biochemical fingerprints. Purity should be evaluated using orthogonal approaches, such as SEC profiling, NTA electron microscopy, and biochemical assays. In addition, potency assays should be tailored to the intended therapeutic indication, including antioxidant, anti-inflammatory, pro-migratory, or regenerative activity where appropriate. Batch-to-batch consistency should be demonstrated across independent production runs using predefined acceptance criteria for particle size, particle concentration, cargo composition, purity, and functional potency.
  Furthermore, allergenicity and immunogenicity should be considered during the quality assessment of PDEVs, especially for formulations derived from edible or medicinal plants. Proteomic profiling may help identify potential plant allergen families, such as profilins, non-specific lipid transfer proteins, and pathogenesis-related proteins. When necessary, additional purification or allergen-mitigation strategies should be evaluated without compromising vesicle integrity or bioactivity. These assessments should follow the general characterization and reporting principles recommended by MISEV2023, while PDEV-specific batch-release criteria remain to be established [235].
  In addition to proteomic screening, glycomic profiling should be considered, given that cross-reactive carbohydrate determinants (CCDs) on plant glycoproteins are a well-established source of false-positive IgE reactivity in allergy diagnostics. Although CCDs seldom cause clinical symptoms, their presence can complicate safety assessments during regulatory reviews. Furthermore, for PDEVs derived from sources of high allergenicity (kiwi, strawberry, peanut), mandatory skin prick equivalence tests using reconstituted PDEV preparations should be included in preclinical safety protocols.
  Scalable production of PDEVs requires isolation technologies that are compatible with large-volume processing. Although DC remains widely used in preclinical studies, its scalability is limited by long processing times, potential vesicle loss or aggregation, co-isolation of soluble proteins, and dependence on ultracentrifugation equipment. In this context, tangential flow filtration (TFF) may provide a scalable approach for concentrating and diafiltering crude plant extracts, while subsequent SEC or other chromatographic purification steps can further reduce soluble proteins and low-molecular-weight contaminants. A TFF-SEC workflow therefore represents a promising GMP-compatible strategy for improving yield, purity, and structural preservation, although process parameters and batch-to-batch reproducibility still require systematic validation for each plant source.
  Concomitantly, the evaluation of the efficacy and safety of PDEV-composites must progress beyond traditional rodent models. Mice and rats predominantly heal through rapid wound contraction, a method that is significantly different from re-epithelialization in human skin, thereby limiting their predictive value for human outcomes. Advanced preclinical models, such as 3D bioengineered skin organoids, are highly recommended. These models accurately replicate the layered epidermal-dermal architecture, human-specific barrier functions, and the complex local immune microenvironments, thus providing a much more reliable prediction of clinical outcomes prior to human trials.
  In addition, porcine full-thickness wound models should be considered as a complementary translational platform due to the close anatomical resemblance between porcine and human skin in aspects such as dermal thickness, hair follicle density, and healing kinetics. Additionally, wound-on-a-chip microfluidic systems that incorporate patient-derived cells could facilitate personalized screening of PDEV-biomaterial composites before clinical application.

## Conclusions

In conclusion, the integration of PDEVs with biomaterials not only capitalizes on the advantages of PDEVs, such as their high bioactivity and superior loading efficiency, but also enhances the excellent biocompatibility and tissue repair properties of biomaterials. This multifunctional combination endows PDEV-biomaterial composites with broad application prospects in wound healing. This review has highlighted the therapeutic advantages of PDEVs in wound healing, outlined the current research progress in biomaterial wound dressings, and comprehensively synthesized studies concerning PDEV-biomaterial composites. It aims to elucidate the synergistic relationships between PDEVs and biomaterials, provide researchers with novel investigative perspectives, and pave the way for advanced therapeutic approaches in wound management.

## Data Availability

Not applicable.
